# Tracking structural evolution: *operando* regenerative CeO_x_/Bi interface structure for high-performance CO_2_ electroreduction

**DOI:** 10.1093/nsr/nwaa187

**Published:** 2020-08-24

**Authors:** Ruichao Pang, Pengfei Tian, Hongliang Jiang, Minghui Zhu, Xiaozhi Su, Yu Wang, Xiaoling Yang, Yihua Zhu, Li Song, Chunzhong Li

**Affiliations:** Key Laboratory for Ultrafine Materials of Ministry of Education, School of Chemical Engineering, East China University of Science and Technology, Shanghai 200237, China; Shanghai Engineering Research Center of Hierarchical Nanomaterials, School of Materials Science and Engineering, East China University of Science and Technology, Shanghai 200237, China; Key Laboratory for Ultrafine Materials of Ministry of Education, School of Chemical Engineering, East China University of Science and Technology, Shanghai 200237, China; Key Laboratory for Ultrafine Materials of Ministry of Education, School of Chemical Engineering, East China University of Science and Technology, Shanghai 200237, China; Key Laboratory for Ultrafine Materials of Ministry of Education, School of Chemical Engineering, East China University of Science and Technology, Shanghai 200237, China; Shanghai Institute of Applied Physics, Chinese Academy of Sciences, Shanghai 201204, China; Shanghai Institute of Applied Physics, Chinese Academy of Sciences, Shanghai 201204, China; Shanghai Engineering Research Center of Hierarchical Nanomaterials, School of Materials Science and Engineering, East China University of Science and Technology, Shanghai 200237, China; Shanghai Engineering Research Center of Hierarchical Nanomaterials, School of Materials Science and Engineering, East China University of Science and Technology, Shanghai 200237, China; National Synchrotron Radiation Laboratory, University of Science and Technology of China, Hefei 230029, China; Key Laboratory for Ultrafine Materials of Ministry of Education, School of Chemical Engineering, East China University of Science and Technology, Shanghai 200237, China; Shanghai Engineering Research Center of Hierarchical Nanomaterials, School of Materials Science and Engineering, East China University of Science and Technology, Shanghai 200237, China

**Keywords:** CO2 electroreduction, electrocatalyst, nanosheet, structural evolution, interface

## Abstract

Unveiling the structural evolution and working mechanism of catalysts under realistic operating conditions is crucial for the design of efficient electrocatalysts for CO_2_ electroreduction, yet remains highly challenging. Here, by virtue of *operando* structural measurements at multiscale levels, it is identified under CO_2_ electroreduction conditions that an as-prepared CeO_2_/BiOCl precatalyst gradually evolves into CeO_x_/Bi interface structure with enriched Ce^3+^ species, which serves as the real catalytically active phase. The derived CeO_x_/Bi interface structure compared to pure Bi counterpart delivers substantially enhanced performance with a formate Faradaic efficiency approaching 90% for 24 hours in a wide potential window. The formate Faradaic efficiency can be further increased by using isotope D_2_O instead of H_2_O. Density functional theory calculations suggest that the regenerative CeO_x_/Bi interfacial sites can not only promote water activation to increase local ^*^H species for CO_2_ protonation appropriately, but also stabilize the key intermediate ^*^OCHO in formate pathway.

## Teaser

*Operando* structural identifications at multiscale levels are employed to track the structural evolution of catalysts under realisticelectrochemical CO_2_ reduction conditions.

## INTRODUCTION

Recent years have witnessed explosive development in electrochemical CO_2_ reduction into valuable chemicals or fuels [1–5]. CO_2_ electroreduction is considered as a promising route to utilizing renewable electricity [6,7]. Designing high-performance electrocatalysts is pivotal to tuning CO_2_ activation, thus achieving the highly selective CO_2_ conversion into target products [8–10]. However, the rational design of electrocatalysts faces severe challenges, because most of the catalysts would go through dynamic structural evolution under applied electric field [1,6,11–13]. The ambiguous evolution rules also hinder the uncovering of the working mechanism. Generally, the real catalytically active phase is inconsistent with the as-prepared or post-catalyzed catalyst structure. The established structure–performance relationship based on *ex situ* static characterizations does not match the realistic catalytic phenomenon. For instance, positive-valence metal species would be electrochemically transformed into so-called zero-valent metals during CO_2_ electroreduction [6,7,14]. In this regard, some studies pointed out that the presence of slight positive-valence metal species in the derived structure is the key to realizing highly efficient CO_2_ electrocatalysis [15–17]. More importantly, the evolution processes are closely related to the precatalytic structure and electrocatalytic conditions, including potentials, electrolytes, temperature, etc [11,12]. Nevertheless, it is not clear how the catalyst structure evolves, and how a real catalytically active component catalyzes CO_2_ conversion under the corresponding environmental conditions [18]. To tackle the problems, *in situ*/*operando* characterization techniques, such as *operando* Raman and X-ray absorption fine structure (XAFS) measurements, are solid methods to track structural change, identify real active phases and uncover the underlying mechanism, thus guiding the structure design of highly active and robust catalysts [19–22].

**Figure 1.**
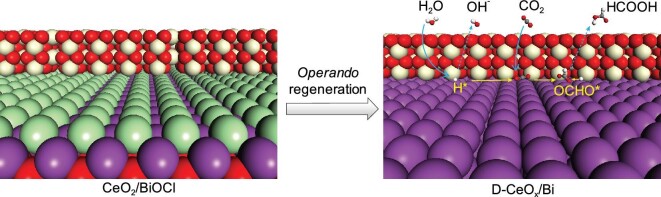
Schematic illustration of the *operando* regeneration of CeO_2_/BiOCl into D-CeO_x_/Bi during CO_2_ electroreduction. Purple, green, yellow, gray, red and white spheres are Bi, Cl, Ce, C, O and H atoms, respectively.

Herein, we *operando* probed the structural evolution of as-prepared BiOCl nanosheets loaded with CeO_2_ (denoted as CeO_2_/BiOCl), which was obviously different from *ex situ* static characterizations either before or after a reaction. It has been found that the CeO_2_/BiOCl precatalyst gradually evolved into a stable CeO_x_/Bi interface structure with increased Ce^3+^ species, under cathodic reduction potential with a certain strength (Fig. 1). The structural regeneration was attributed to the irreversible reduction processes of Bi^3+^ and Ce^4+^ cations. Especially, the presence of CeO_2_ component could facilitate the regeneration process, as evidenced by *operando* Raman results. The derived CeO_x_/Bi interface structure (named as D-CeO_x_/Bi) enabled highly selective and stable CO_2_ electroreduction to formate, significantly outperforming the Bi and CeO_x_ (denoted as D-Bi and D-CeO_x_) counterparts derived from cathodic reduction of BiOCl and CeO_2_. D_2_O isotope labeling experiments have also been performed to probe the working mechanism, verifying that hydrogen source of formate product originated from water activation. Based on the *operando* structural identifications, the structure model for the regenerated Bi/CeO_x_ interface structure was established. The computed Gibbs free energies of the reaction species indicated that the formation of key intermediate ^*^OCHO for formate pathway could be boosted at the regenerative CeO_x_/Bi interfacial sites, thereby rationalizing the high selectivity experimentally.

## RESULTS AND DISCUSSION

**Figure 2.**
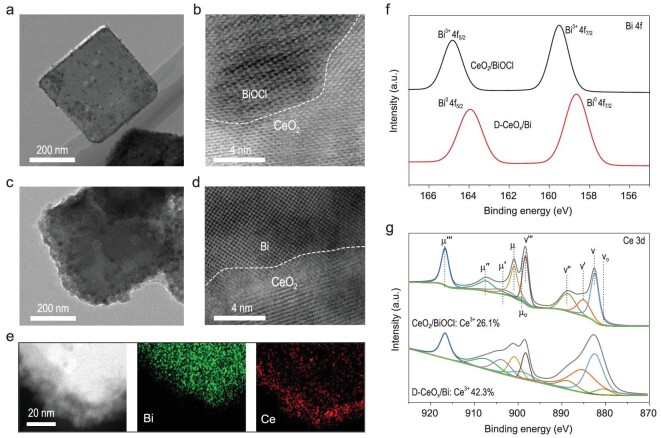
*Ex situ* static characterizations before and after the reaction. (a) TEM and (b) HRTEM images of the CeO_2_/BiOCl. (c) TEM image, (d) HRTEM image and (e) the corresponding EDX elemental mapping of the D-CeO_x_/Bi. (f) Bi 4f and (g) Ce 3d XPS spectra of the CeO_2_/BiOCl and D-CeO_x_/Bi.

The BiOCl and CeO_2_/BiOCl were obtained via a propylene oxide-assisted hydrolytic process, followed by thermal treatment at 400°C in air (see details in Methods). X-ray diffraction (XRD) measurements clarified that both BiOCl and CeO_2_ components existed in the CeO_2_/BiOCl (Supplementary Fig. 1). The CeO_2_ nanoparticles (NP) were loaded onto typical BiOCl nanosheets to form interfacial structure, as confirmed by transmission electron microscopy (TEM) and corresponding fast Fourier Transform images (Fig. 2a and b, Supplementary Figs 2–4). Subjected to a cathodic reduction at −1.0 V versus reversible hydrogen electrode (RHE) for 30 minutes in CO_2_-saturated 0.5 M KHCO_3_ solution, slight structure change was observed for the D-CeO_x_/Bi (Fig. 2c), probably due to the irreversible reduction of Bi^3+^ and Ce^4+^ cations [6,7,14]. The NP-loaded interfacial structure was maintained (Fig. 2d). As shown in Supplementary Fig. 5, the lattice spacing of 0.33 and 0.27 nm in the D-CeO_x_/Bi were attributed to the Bi (012) and CeO_2_ (110) planes (JCPDS No. 44-1246: Bi; JCPDS No. 34-0394: CeO_2_), respectively [6,23,24]. The results indicated that the BiOCl was converted into metallic Bi, and the CeO_2_ was apparently unchanged. We also identified that the two phases kept close interfacial contact from high angle annular dark field scanning TEM (HAADF-STEM) and the corresponding energy-dispersive X-ray (EDX) elemental mapping (Fig. 2e). Furthermore, the transformation process was also evidenced by X-ray photoelectron spectroscopy (XPS) results. The peaks of 164.8 and 159.5 eV corresponding to Bi^3+^ species disappeared (Fig. 2f), and two new peaks at 163.9 and 158.6 eV ascribed to metallic Bi^0^ were detected after the reduction process [14]. The Ce species kept typical signal peaks corresponding to Ce^3+^ and Ce^4+^ species both before and after the reduction (Fig. 2g). It is noted that the Ce^3+^ species were obviously enriched from 26.1% to 42.3% as a result of the increase of oxygen defects during the reduction process (Supplementary Table 1) [24]. And these oxygen defects were readily filled by the hydroxyl in aqueous solution [25]. *Ex situ* Raman measurements were also performed to characterize the D-CeO_x_/Bi. As shown in Supplementary Fig. 6, for the CeO_x_ sample, the peak at 1168 cm^−1^ corresponded to the second-order longitudinal optical (2LO) mode of fluorite phase [26]. As to D-CeO_x_/Bi, the peaks at 75 and 103 cm^−1^ corresponded to typical vibration signal of metallic Bi [27]. Compared to that of CeO_x_ sample, a new peak at 1071 cm^−1^ emerged for the D-CeO_x_/Bi, which was ascribed to the formation of Bi-O-Ce bonds [28,29]. The Raman results also indicated that Bi^3+^ of BiOCl was reduced to Bi^0^ species, and Bi/CeO_x_ interfaces existed in the D-CeO_x_/Bi. The standard electrode potential of *E*^0^ (Bi^3+^/Bi^0^) is 0.308 V versus RHE [30], much more positive than the cathodic reduction potential of −1.0 V versus RHE. Therefore, the BiOCl was readily converted into metallic Bi. The *E*^0^ (Ce^3+^/Ce^0^) is −2.336 V versus RHE, and *E*^0^ (CeO_2_/Ce^3+^) is 1.4 V versus RHE. Thus Ce^4+^ species in the CeO_2_ component were readily reduced to the Ce^3+^ species, but were hardly converted to Ce^0^ species. It has to be pointed out that nanostructured metallic Bi is air-sensitive, and is easily surface-oxidized [24]. And the CeO_2_ has the high ability to capture and release oxygen [31,32]. Therefore, combined with the above *ex situ* static characterizations, *in situ*/*operando* structural measurements have to be employed to identify the real active components accurately.

**Figure 3.**
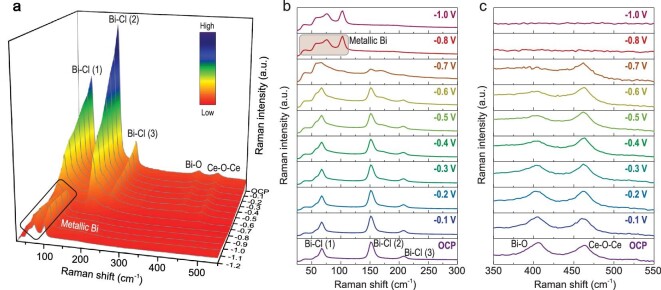
*Operando* Raman characterizations towards regeneration process. (a) Potential-dependent *operando* Raman spectra of the CeO_2_/BiOCl. Potential-dependent *operando* Raman spectra of the CeO_2_/BiOCl at wavenumber region of 25 to 300 cm^−1^ (b) and 350 to 550 cm^−1^ (c), respectively.

We carried out *operando* characterization measurements at multiscale levels to track the structural evolution and obtain dynamic structural information of the catalysts in CO_2_-saturated 0.5 M KHCO_3_ solution. First *operando* XRD measurement was performed to identify the evolution of crystal structure. Typically, from the *operando* XRD patterns (Supplementary Fig. 7), it can be seen that the peak of about 2θ = 25.9° corresponded to the (101) plane of BiOCl. When the cathodic reduction potential was increased to −0.9 V versus RHE, the peak disappeared. The new peak at about 2θ = 27.2° corresponding to the (012) plane of metallic Bi was emerged. The peak at about 2θ = 26.6° ascribed to carbon paper nearly remained unchanged. The signal from CeO_2_ component was not detected in the *operando* test condition due to the relatively low content. The transformation of crystal structure was in accordance with the above TEM results. To achieve the identification of the dynamic geometric structure, we performed potential- and time-dependent *operando* Raman spectra (Fig. 3, Supplementary Figs 8 and 9). At open circuit potential (OCP), four typical Raman peaks at 67, 151, 207 and 405 cm^−1^ were presented (Fig. 3), which were assigned to the A_1g_ external Bi-Cl stretching mode, the A_1g_ internal Bi-Cl stretching mode, the E_g_ external Bi-Cl stretching mode and the external Bi-O stretching vibration in the BiOCl structure, respectively [33,34]. The peak at 461 cm^−1^ corresponded to the F_2g_ mode of Ce-O-Ce symmetric stretching vibration in the CeO_2_ structure [35]. With gradually increasing cathodic potential to −0.7 V versus RHE, the peaks ascribed to that of the BiOCl were in decline (Fig. 3a and b). Meanwhile, it was accompanied by two new characteristic peaks at 75 and 103 cm^−1^, which were corresponding to typical vibration signal of metallic Bi [27]. Also note that the peak ascribed to the F_2g_ mode of Ce-O-Ce symmetric stretching vibration also remained almost unchanged until −0.7 V (Fig. 3c). The peak vanished at higher potentials. With the increase of oxygen defects and reduced Ce^3+^ ions at higher potentials, the signature vibrational band would exhibit a reduced and widened model system [26,36], thus leading to the apparent disappearance of the peak at about 461 cm^−1^. Furthermore, we also dissected *operando* Raman spectra from 950 to 1200 cm^−1^ (Supplementary Fig. 8). The peak of about 1071 cm^−1^ remained nearly unchanged under applied potential windows, manifesting in the persistence of the Bi/CeO_x_ interface throughout electrochemical tests. Additionally, by analyzing time-dependent *operando* Raman spectra at −0.9 V versus RHE (Supplementary Fig. 9), it was also found that the CeO_2_/BiOCl compared to the BiOCl exhibited faster transformation process, probably because the presence of the CeO_2_ could facilitate water dissociation to increase local proton concentration for the BiOCl reduction [37]. To further gain the information for electronic properties, we employed *operando* X-ray absorption near edge structure (XANES) measurements. From the normalized XANES spectra and local enlargement at the Bi L_3_-edge (Supplementary Fig. 10), it was found that the BiOCl subjected to the cathodic reduction current of 1 and 10 mA/cm^2^ aligned well with the Bi foil, and the shift of absorption edge could not be observed, demonstrating the complete absence of positive-valence Bi species [6,14]. The normalized Ce L_3_-edge XANES spectra (Supplementary Fig. 11) show that the Ce species of CeO_2_/BiOCl was almost reduced to Ce^3+^ under CO_2_ electroreduction conditions [35]. The result is inconsistent with Ce 3d XPS results as a result of the high ability of oxygen capture for oxygen vacancy-rich CeO_x_ in air [31,32]. These *operando* characterization results provided solid evidences for the structural regeneration of the as-prepared CeO_2_/BiOCl precatalyst into the defect-enriched CeO_x_/Bi interface structure under the actual CO_2_ electroreduction condition.

**Figure 4.**
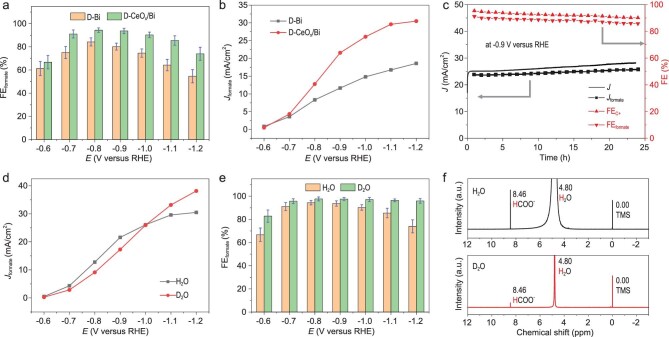
Performance evaluation of CO_2_ electroreduction. (a) Potential-dependent FE_formate_ of the D-Bi and D-CeO_x_/Bi. (b) Potential-dependent formate partial current density of the D-Bi and D-CeO_x_/Bi. (c) Long-term stability and selectivity of D-CeO_x_/Bi at −0.9 V versus RHE. (d) Potential-dependent formate partial current density of the D-CeO_x_/Bi under H_2_O and D_2_O conditions. (e) Potential-dependent FE_formate_ of the D-CeO_x_/Bi under H_2_O and D_2_O conditions. (f) ^1^H nuclear magnetic resonance (NMR) spectra of the D-CeO_x_/Bi under H_2_O and D_2_O conditions.

The electrochemical performance was estimated in a two-compartment gas-tight H-type cell within 0.5 M KHCO_3_ solution (see details in Methods). In order to accomplish the structural regeneration, first the as-prepared CeO_2_/BiOCl and BiOCl loaded onto carbon paper were electrochemically treated for 30 minutes at −0.9 V versus RHE. The linear sweep voltammetry (LSV) curves (Supplementary Fig. 12) displayed that the current densities were remarkably increased in CO_2_-saturated electrolyte in contrast to Ar-saturated electrolyte, indicating that both the D-CeO_x_/Bi and D-Bi favored CO_2_ reduction over hydrogen evolution reaction (HER). Through analyses of the products, the D-CeO_x_/Bi with Bi/Ce ratio (3 : 1) exhibited the optimized performance, displaying the highest formate Faradaic efficiency (FE_formate_) of 92.0% at −0.9 V versus RHE (Fig. 4a, Supplementary Figs 13 and 14). The corresponding formate partial current density reached 22.1 mA/cm^2^ (Fig. 4b), which was nearly two times higher than that of the D-Bi (12.0 mA/cm^2^). The electrocatalytic performance of both D-Bi and D-CeO_x_/Bi is significantly better than of D-CeO_x_ (Supplementary Fig. 15), evidencing the importance of Bi components on high formate selectivity [6,7,14]. The electrochemical surface area (ECSA)-normalized partial current density of D-CeO_x_/Bi was still 1.5 times larger than that of D-Bi (Supplementary Fig. 16), suggesting the key role of the interfacial structure on enhancing intrinsic activity [25,35]. We also identified that the FE of products that contain carbon element (formate and CO) maintained over 90% at a wide potential window of approximately −0.7 to −1.0 V (Supplementary Fig. 17). In addition to the good activity and selectivity, the D-CeO_x_/Bi also displayed long-term stability of formate production for 24 hours (Fig. 4c).

To probe the underlying mechanism of CO_2_ electroreduction into formate onto the D-CeO_x_/Bi, we carried out *operando* Fourier transformation infrared (FTIR) measurements and D_2_O labeling experiments. As shown in Supplementary Fig. 18a, the broad band between 1350 and 1450 cm^−1^ arose from the dissolved CO_3_^2−^/HCO_3_^−^ and formate [38–40]. The broad band gradually rose with increasing reduction potential, suggesting the accumulation of formate [38]. The reversed peak at about 1650 cm^−1^ is attributed to the consumption of interfacial H_2_O [41,42], implying the H_2_O participation in the formate formation. Meanwhile, the

doublet peak at about 2350 cm^−1^ declined when the cathodic reduction potential or the electrolytic time was increased (Supplementary Fig. 18b and c), which are attributed to the consumption of gaseous CO_2_ [43]. From potential-dependent current densities of the D-CeO_x_/Bi under H_2_O and D_2_O conditions (Fig. 4d, Supplementary Fig. 19), we could identify that the partial current densities of total formate (HCOO^−^ and DCOO^−^) under D_2_O condition were lower than that under H_2_O condition when the potential was between −0.6 and −1.0 V. For the potential window of −1.0 to −1.2 V, the partial current densities under D_2_O condition were larger relative to that under H_2_O condition. The FE_formate_ under D_2_O condition was always higher than that under H_2_O condition under total measured potential window (Fig. 4e, Supplementary Fig. 20). The changed activity and selectivity meant that the formate formation was moderately promoted, and the hydrogen evolution was inhibited under D_2_O condition. To disclose the reason, the HCOO^−^ content in the total electrolyte was determined by ^1^H nuclear magnetic resonance (NMR) (Fig. 4f). Through analyzing the relative amount of HCOO^−^ and DCOO^−^ in the total formate (see details in Methods), it was uncovered that the DCOO^−^ content of 91.7% at −0.9 V was much greater than the HCOO^−^ content, which demonstrated that the hydrogen source of formate mainly came from water activation [44]. Therefore, it could be easily understood that the water activation under D_2_O condition was harder than that under H_2_O condition, causing the insufficient supply of absorbed ^*^D species for the formate formation at low overpotentials. At high overpotentials, the supply of the absorbed ^*^D species was sufficient. The appropriately inhibited water activation enabled less release of gaseous hydrogen, thus leading to the higher formate partial current densities and selectivity.

**Figure 5.**
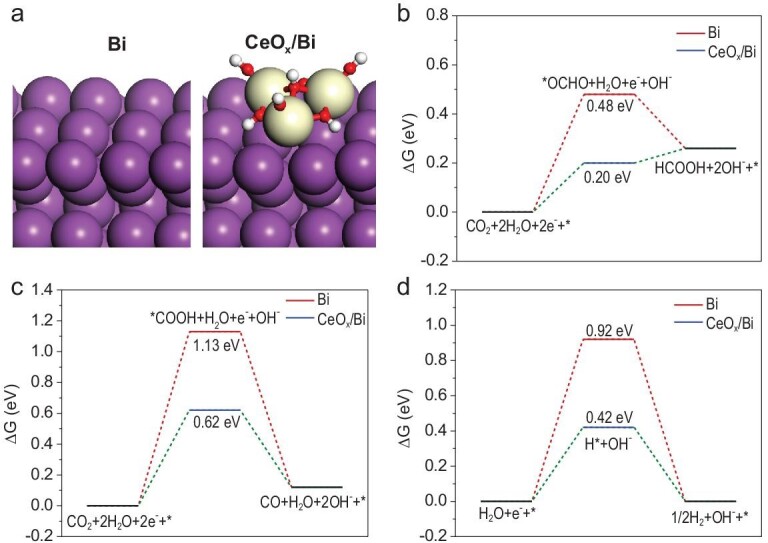
Theoretical calculations for the Bi and CeO_x_/Bi. (a) Optimized structure models for the Bi and CeO_x_/Bi. Purple, yellow, red and white spheres are Bi, Ce, O, and H atoms, respectively. (b, c) Gibbs free energy diagrams for CO_2_RR to HCOOH and CO onto the Bi and CeO_x_/Bi structure models, respectively. (d) Gibbs free energy diagrams for H_2_O activation onto the Bi and CeO_x_/Bi structure models.

We further employed density functional theory (DFT) calculations to understand the enhanced reason of the regenerated Ce^3+^-enriched CeO_x_/Bi interface toward formate formation. Based on the above *operando* structural identifications, structure models for the Bi and CeO_x_/Bi surfaces as well as corresponding adsorption configurations of intermediates were rationally established (Fig. 5a and Supplementary Fig. 21). In the structure models, the Ce^3+^ species were considered as the Ce atoms that were bound with hydroxyls [25]. The formation of ^*^OCHO and ^*^COOH intermediate were demonstrated as the key of HCOOH and CO pathway, respectively [45,46]. For the HCOOH pathway, the Gibbs free energies for the formation of ^*^OCHO onto Bi and CeO_x_/Bi surfaces were 0.48 and 0.20 eV, respectively (Fig. 5b). The introduction of CeO_2_ onto Bi surface efficiently stabilized the ^*^OCHO through binding one O atom of ^*^OCHO with the Ce atom (Supplementary Fig. 21c). On the other hand, the calculated Gibbs free energies for CO pathway onto both the Bi and CeO_x_/Bi surfaces were larger than that for HCOOH pathway (Fig. 5c), strongly supporting higher selectivity toward formate than that toward CO onto typical Bi-based materials. To understand the effect of CeO_x_/Bi interface on water activation, we also calculated the Gibbs free energies of HER onto Bi and CeO_x_/Bi surfaces (Fig. 5d). The Gibbs free energies for the ^*^H formation onto the Bi surface were 0.92 eV. The value for the CeO_x_/Bi surface was remarkably decreased to 0.42 eV. The results indicated that water activation could be efficiently enhanced through introducing CeO_2_ components onto the Bi surface. In terms of the competition between HER and HCOOH formation, it could be identified that the formation energies of the key intermediate ^*^OCHO were always lower than that of ^*^H, theoretically interpreting the preferential HCOOH formation onto the CeO_x_/Bi interfacial structure, despite the boosted water activation. Combining these calculation results with the above isotope labeling evidences, it could be reasonably deduced that the water activation at the interfacial sites was properly improved to provide the ^*^H species for the formation of the key intermediate ^*^OCHO in HCOOH pathway, thus accounting for the increased selectivity of formate experimentally.

## CONCLUSION

In summary, we have reported the tracking of the dynamic structural evolution of as-prepared CeO_2_/BiOCl precatalyst under realistic CO_2_ electroreduction conditions, by combining *operando* XRD, Raman and XAFS measurements. It has comprehensively been demonstrated that the real active catalyst for CO_2_ reduction is regenerative CeO_x_/Bi interface structure with increased Ce^3+^ species. High Faradaic efficiency and stability for formate formation are finally exhibited onto the derived CeO_x_/Bi interface structure. D_2_O isotope labeling results have verified that the hydrogen elements of formate product originate from water activation. We have revealed that the regenerative CeO_x_/Bi interfacial sites can properly promote water activation to supply hydrogen source for the formate formation, and lower the energy barrier of the CO_2_ protonation into the key intermediate ^*^OCHO. This work provides insights into structural evolution and activity origin of catalysts under realistic working conditions, and highlights the importance of mechanism study and catalyst design based *operando* feedback information.

## METHODS

### Synthesis of CeO_2_/BiOCl and BiOCl

CeO_2_/BiOCl was synthesized through a propylene oxide (PO)-assisted hydrolytic process. Typically, BiCl_3_ (1.5 mmol) and CeCl_3_$\cdot$7H_2_O (0.5 mmol) were firstly dissolved in deionized water (4.5 ml). Then PO (1.3 ml) was added in the above liquid to obtain the liquid emulsion. The solution was stirred for 6 hours and then aged for 6 hours. The obtained precipitate was collected by centrifuging and washed five times with deionized water and ethanol, then dried at 60°C for 8 hours in the air. Finally the obtained product was placed into a muffle furnace and heated to 400°C for 2 hours at a rate of 3°C/min. BiOCl counterpart was prepared with the similar method except that CeCl_3_$\cdot$7H_2_O was not added.

### Electrochemical measurements

Electrochemical measurements were performed in a three-electrode two-compartment cell separated by Nafion 117 membrane in an electrochemical workstation (CHI 660E). ^1^H NMR spectroscopy was used to analyze liquid products. The ^1^H NMR spectra were recorded on an Ascend 600-MHz Unity plus spectrometer (Bruker). The electrolyte (0.5 mL) was mixed with deuterated water (0.1 mL), using 1% tetramethylsilane (TMS) as internal standard. CO_2_RR measurements in D_2_O solution were carried out with similar processes to that in H_2_O. The total amount of formate (HCOO^−^ and DCOO^−^) in electrolytes was quantified by High Performance Liquid Chromatography (HPLC). ^1^H NMR was used to determine the amount of HCOO^−^ product. The concentration of formate was quantitatively determined from its NMR peak area relative to the internal standard by using the calibration curve for standard HCOOK solutions (Supplementary Fig. 22). *Operando* XRD experiments were performed using an X-Ray Polycrystalline Diffractometer (Bruker AXS D8 Advance). *Operando* Raman measurements were conducted in a LabRAM HR800 confocal microscope (Horiba Jobin Yvon). *Operando* XAFS measurements were performed in Shanghai Synchrotron Radiation Facility (BL14W1, SSRF), China. The *operando* FTIR experiments were performed with Perkin Elmer Spectrum 100 FT-IR spectrometer equipped with diamond attenuated total reflection (UATR) accessory and a mercury cadmium telluride (MCT) detector. More electrochemical and *operando* measurement details were given in the supplementary information.

### DFT calculation

DFT calculations were performed using the Vienna ab-initio simulation package (VASP) [47,48]. Considering that the CO_2_RR was carried out at aqueous phase condition, a fully hydroxylated ceria cluster (Ce_3_O_7_H_7_) supported on Bi(110) surface is adopted to simulate the D-CeO_x_/Bi catalyst, denoted as Ce_3_O_7_H_7_/Bi(110) [49]. Bi(110) surface was adopted to model the Bi catalyst for reference. Bi(110) substrate was established as a four-layered slab with (3 × 3) unit cells, of which the bottom two layers were fixed. The top two layers of Bi(110), the Ce_3_O_7_H_7_ cluster and the surface species were fully relaxed. A vacuum spacing of 12 Å along the normal direction (z) to the surface and a 1 × 2 × 1 Gamma centered k-point sampling were used for the models. More computational details were given in the supplementary information.

## Supplementary Material

nwaa187_Supplemental_File

## References

[1] Wu Y , Jiang Z, Lu X et al. Domino electroreduction of CO_2_ to methanol on a molecular catalyst. Nature 2019; 575: 639–42. 10.1038/s41586-019-1760-8 31776492

[2] Zhang L , Zhao Z, Wang T et al. Nano-designed semiconductors for electro- and photoelectro-catalytic conversion of carbon dioxide. Chem Soc Rev 2018; 47: 5423–43. 10.1039/C8CS00016F 29799046

[3] Su X , Yang X-F, Huang Y et al. Single-atom catalysis toward efficient CO_2_ conversion to CO and formate products. Acc Chem Res 2019; 52: 656–64. 10.1021/acs.accounts.8b00478 30512920

[4] Ren W , Zhao C. Paths towards enhanced electrochemical CO_2_ reduction. Natl Sci Rev 2020; 7: 7–9. 10.1093/nsr/nwz121 PMC8289043 34692011

[5] Han N , Wang Y, Ma L et al. Supported cobalt polyphthalocyanine for high-performance electrocatalytic CO_2_ reduction. Chem 2017; 3: 652–64. 10.1016/j.chempr.2017.08.002

[6] Gong Q , Ding P, Xu M et al. Structural defects on converted bismuth oxide nanotubes enable highly active electrocatalysis of carbon dioxide reduction. Nat Commun 2019; 10: 2807. 10.1038/s41467-019-10819-4 31243275 PMC6594929

[7] Han N , Wang Y, Yang H et al. Ultrathin bismuth nanosheets from *in situ* topotactic transformation for selective electrocatalytic CO_2_ reduction to formate. Nat Commun 2018; 9: 1320. 10.1038/s41467-018-03712-z 29615621 PMC5882965

[8] Ma W , Xie S, Liu T et al. Electrocatalytic reduction of CO_2_ to ethylene and ethanol through hydrogen-assisted C-C coupling over fluorine-modified copper. Nat Catal 2020, 3: 478–87. 10.1038/s41929-020-0450-0

[9] Liu M , Liu M, Wang X et al. Quantum-dot-derived catalysts for CO_2_ reduction reaction. Joule 2019; 3: 1703–18. 10.1016/j.joule.2019.05.010

[10] Liu M , Pang Y, Zhang B et al. Enhanced electrocatalytic CO_2_ reduction via field-induced reagent concentration. Nature 2016; 537: 382–6. 10.1038/nature19060 27487220

[11] Jiang H , He Q, Li X et al. Tracking structural self-reconstruction and identifying true active sites toward cobalt oxychloride precatalyst of oxygen evolution reaction. Adv Mater 2019; 31: 1805127. 10.1002/adma.201805127 30633404

[12] Jiang H , Lin Y, Chen B et al. Ternary interfacial superstructure enabling extraordinary hydrogen evolution electrocatalysis. Mater Today 2018; 21: 602–10. 10.1016/j.mattod.2018.01.033

[13] Liang Y , Zhou W, Shi Y et al. Unveiling *in situ* evolved In/In_2_O_3-x_ heterostructure as the active phase of In_2_O_3_ toward efficient electroreduction of CO_2_ to formate. Sci Bull 2020; 65: 1547–54. 10.1016/j.scib.2020.04.022 36738072

[14] He S , Ni F, Ji Y et al. The p-orbital delocalization of main-group metals to boost CO_2_ electroreduction. Angew Chem Int Ed 2018; 57: 16114–9. 10.1002/anie.201810538 30315718

[15] Chen Y , Kanan MW. Tin oxide dependence of the CO_2_ reduction efficiency on tin electrodes and enhanced activity for tin/tin oxide thin-film catalysts. J Am Chem Soc 2012; 134: 1986–9. 10.1021/ja2108799 22239243

[16] De Luna P , Quintero-Bermudez R, Dinh C-T et al. Catalyst electro-redeposition controls morphology and oxidation state for selective carbon dioxide reduction. Nat Catal 2018; 1: 103–10. 10.1038/s41929-017-0018-9

[17] Lei Q , Zhu H, Song K et al. Investigating the origin of enhanced C_2+_ selectivity in oxide-/hydroxide-derived copper electrodes during CO_2_ electroreduction. J Am Chem Soc 2020; 142: 4213–22. 10.1021/jacs.9b11790 32041401

[18] Deng P , Wang H, Qi R et al. Bismuth oxides with enhanced bismuth-oxygen structure for efficient electrochemical reduction of carbon dioxide to formate. ACS Catal 2020; 10: 743–50. 10.1021/acscatal.9b04043

[19] Ye K , Zhou Z, Shao J *et al.* *In situ* reconstruction of a hierarchical Sn-Cu/SnO_x_ core/shell catalyst for high-performance CO_2_ electroreduction. Angew Chem Int Ed 2020; 59: 4814–21. 10.1002/anie.201916538 31944516

[20] Zhu Y , Wang J, Chu H *et al.* *In situ*/*operando* studies for designing next-generation electrocatalysts. ACS Energy Lett 2020; 5: 1281–91. 10.1021/acsenergylett.0c00305

[21] Jiang H , He Q, Zhang Y et al. Structural self-reconstruction of catalysts in electrocatalysis. Acc Chem Res 2018; 51: 2968–77. 10.1021/acs.accounts.8b00449 30375841

[22] Wang Y , Zhou W, Jia R et al. Unveiling the activity origin of a copper-based electrocatalyst for selective nitrate reduction to ammonia. Angew Chem Int Ed 2020; 59: 5350–4. 10.1002/anie.201915992 31965695

[23] Zhang S , Xia Z, Zou Y et al. Interfacial frustrated Lewis pairs of CeO_2_ activate CO_2_ for selective tandem transformation of olefins and CO_2_ into cyclic carbonates. J Am Chem Soc 2019; 141: 11353–7. 10.1021/jacs.9b03217 31290659

[24] Lin Y , Yang L, Jiang H et al. Sulfur atomically doped bismuth nanobelt driven by electrochemical self-reconstruction for boosted electrocatalysis. J Phys Chem Lett 2020; 11: 1746–52. 10.1021/acs.jpclett.0c00134 32048849

[25] Dong H , Zhang L, Li L et al. Abundant Ce^3+^ ions in Au-CeO_x_ nanosheets to enhance CO_2_ electroreduction performance. Small 2019; 15: 1900289. 10.1002/smll.201900289 30938486

[26] Lin B , Liu Y, Heng L et al. Morphology effect of ceria on the catalytic performances of Ru/CeO_2_ catalysts for ammonia synthesis. Ind Eng Chem Res 2018; 57: 9127–35. 10.1021/acs.iecr.8b02126

[27] Zhang W , Hu Y, Ma L et al. Liquid-phase exfoliated ultrathin Bi nanosheets: uncovering the origins of enhanced electrocatalytic CO_2_ reduction on two-dimensional metal nanostructure. Nano Energy 2018; 53: 808–16. 10.1016/j.nanoen.2018.09.053

[28] Satsuma A , Yanagihara M, Ohyama J et al. Oxidation of CO over Ru/Ceria prepared by self-dispersion of Ru metal powder into nano-sized particle. Catal Today 2013; 201: 62–7. 10.1016/j.cattod.2012.03.048

[29] Huang W , Gao Y. Morphology-dependent surface chemistry and catalysis of CeO_2_ nanocrystals. Catal Sci Technol 2014; 4: 3772–84. 10.1039/C4CY00679H

[30] Lide DR . CRC Handbook of Chemistry and Physics, 87th edn. Boca Raton: CRC Press/Taylor and Francis Group, 2006.

[31] Hu Q , Huang B, Li Y et al. Methanol gas detection of electrospun CeO_2_ nanofibers by regulating Ce^3+^/Ce^4+^ mole ratio via Pd doping. Sens Actuators B Chem 2020; 307: 127638. 10.1016/j.snb.2019.127638

[32] Michel CR , Martínez-Preciado AH. CO sensor based on thick films of 3D hierarchical CeO_2_ architectures. Sens Actuators B Chem 2014; 197: 177–84. 10.1016/j.snb.2014.02.090

[33] Davies JED . Solid state vibrational spectroscopy-III[1] The infrared and raman spectra of the bismuth(III) oxide halides. J Inorg Nucl Chem 1973; 35: 1531–4. 10.1016/0022-1902(73)80242-8

[34] Lei Y , Wang G, Song S et al. Synthesis, characterization and assembly of BiOCl nanostructure and their photocatalytic properties. CrystEngComm 2009; 11: 1857–62. 10.1039/b909013b

[35] Lee CW , Shin S-J, Jung H et al. Metal-oxide interfaces for selective electrochemical C-C coupling reactions. ACS Energy Lett 2019; 4: 2241–8. 10.1021/acsenergylett.9b01721

[36] Schilling C , Hofmann A, Hess C et al. Raman spectra of polycrystalline CeO_2_: a density functional theory study. J Phys Chem C 2017; 121: 20834–49. 10.1021/acs.jpcc.7b06643

[37] Liu Z , Huang E, Orozco I et al. Water-promoted interfacial pathways in methane oxidation to methanol on a CeO_2_-Cu_2_O catalyst. Science 2020; 368: 513–7. 10.1126/science.aba5005 32355028

[38] Gao D , Zhou H, Cai F et al. Switchable CO_2_ electroreduction via engineering active phases of Pd nanoparticles. Nano Res 2017; 10: 2181–91. 10.1007/s12274-017-1514-6

[39] Yang Y-Y , Ren J, Zhang H-X et al. Infrared spectroelectrochemical study of dissociation and oxidation of methanol at a palladium electrode in alkaline solution. Langmuir 2013; 29: 1709–16. 10.1021/la305141q 23311730

[40] Miyake H , Okada T, Samjeské G et al. Formic acid electrooxidation on Pd in acidic solutions studied by surface-enhanced infrared absorption spectroscopy. Phys Chem Chem Phys 2008; 10: 3662–9. 10.1039/b805955a 18563227

[41] Jiang K , Xu K, Zou S et al. B-Doped Pd catalyst: boosting room-temperature hydrogen production from formic acid-formate solutions. J Am Chem Soc 2014; 136: 4861–4. 10.1021/ja5008917 24635163

[42] Zhang H-X , Wang S-H, Jiang K *et al.* *In situ* spectroscopic investigation of CO accumulation and poisoning on Pd black surfaces in concentrated HCOOH. J Power Sources 2012; 199: 165–9. 10.1016/j.jpowsour.2011.10.033

[43] Yuan T , Hu Z, Zhao Y et al. Two-dimensional amorphous SnO_x_ from liquid metal: mass production, phase transfer, and electrocatalytic CO_2_ reduction toward formic acid. Nano Lett 2020; 20: 2916–22. 10.1021/acs.nanolett.0c00844 32155077

[44] Ma W , Xie S, Zhang X-G et al. Promoting electrocatalytic CO_2_ reduction to formate via sulfur-boosting water activation on indium surfaces. Nat Commun 2019; 10: 892. 10.1038/s41467-019-08805-x 30792388 PMC6385284

[45] Kortlever R , Shen J, Schouten KJP et al. Catalysts and reaction pathways for the electrochemical reduction of carbon dioxide. J Phys Chem Lett 2015; 6: 4073–82. 10.1021/acs.jpclett.5b01559 26722779

[46] Yoo JS , Christensen R, Vegge T et al. Theoretical insight into the trends that guide the electrochemical reduction of carbon dioxide to formic acid. ChemSusChem 2016; 9: 358–63. 10.1002/cssc.201501197 26663854

[47] Kresse G , Furthmüller J. Efficient iterative schemes for total-energy calculations using a plane-wave basis set. Phys Rev B 1996; 54: 11169–86. 10.1103/PhysRevB.54.11169 9984901

[48] Kresse G , Furthmüller J. Efficiency of ab-initio total energy calculations for metals and semiconductors using a plane-wave basis set. Comput Mater Sci 1996; 6: 15–50. 10.1016/0927-0256(96)00008-0 9984901

[49] Gao D , Zhang Y, Zhou Z et al. Enhancing CO_2_ electroreduction with the metal-oxide interface. J Am Chem Soc 2017; 139: 5652–5. 10.1021/jacs.7b00102 28391686

