# Supplementary material for: Tracking structural evolution: operando regenerative CeOx/Bi interface structure for high-performance CO2 electroreduction
Source: Natl Sci Rev. 2020 Aug 24;8(7):nwaa187. doi: 10.1093/nsr/nwaa187 (PMC8310765; doi:10.1093/nsr/nwaa187)
Supplement: nwaa187_Supplemental_File [file nwaa187_supplemental_file.docx]

**Supplementary information for**

**Tracking structural evolution: *operando* regenerative CeO_x_/Bi interface structure for high-performance CO_2_ electroreduction**

Ruichao Pang^1,2^, Pengfei Tian^1^, Hongliang Jiang^1^*****, Minghui Zhu^1^, Xiaozhi Su^3^, Yu Wang^3^, Xiaoling Yang^2^, Yihua Zhu^2^, Li Song^4^, Chunzhong Li^1,2^*****

^1^Key Laboratory for Ultrafine Materials of Ministry of Education, School of Chemical Engineering, East China University of Science and Technology, Shanghai 200237, China

^2^Shanghai Engineering Research Center of Hierarchical Nanomaterials, School of Materials Science and Engineering, East China University of Science and Technology, Shanghai 200237, China

^3^Shanghai Institute of Applied Physics, Chinese Academy of Sciences, Shanghai, 201204, China

^4^National Synchrotron Radiation Laboratory, University of Science and Technology of China, Hefei, Anhui 230029, China

Ruichao Pang and Pengfei Tian contributed equally to this work.

*****Correspondence to: jhlworld@ecust.edu.cn (Hongliang Jiang); czli@ecust.edu.cn (Chunzhong Li)

**Materials and Methods**

**Chemicals.** Bismuth trichloride (BiCl_3_) and Cerium (Ⅲ) chloride heptahydrate (CeCl_3_·7H_2_O) were purchased from Shanghai Macklin Biochemical Co., Ltd. 1,2-Epoxypropane (PO) was purchased from Shanghai Titan Technology Co., Ltd. Nafion solution (5 wt%) and Nafion 117 membrane were obtained from Sigma-Aldrich. Carbon fiber paper was purchased from Toray. Deuterium oxide (D_2_O) was purchased from Shanghai Aladdin Biochemical Technology Co., Ltd. Potassium bicarbonate (KHCO_3_) was purchased from Tianjin Damao Chemical Reagent Co., Ltd. All the chemicals were of analytical grade.

**Characterization.** Transmission electron microscopy (TEM) was carried out on a Jeol JEM-2100 microscope operating at 200 kV. All samples for TEM measurements were ultrasonically dispersed in ethanol and drop cast onto Cu grids covered with a carbon film. X-ray powder diffraction (XRD) equipped with a Philips X`Pert Pro Super diffractometer equipped with Cu Kα radiation (λ=1.54178 Å) was used for powder analysis. The outcomes of X-ray photoelectron spectroscopy (XPS) were acquired with VG ESCA 2000 with an Mg anode. All data were corrected using the C 1s peak at 284.6 eV as an internal standard. The high-angle annular dark-field scanning transmission electron microscopy (HAADF-STEM) was performed on a JEOL JEM-ARF300F TEM/STEM with a spherical aberration corrector. The Bi *L_3_*-edge XAFS spectra were collected at Shanghai Synchrotron Radiation Facility (BL14W1, SSRF), China. Raman spectroscopic measurements were performed using a LabRAM HR800 confocal microscope (Horiba Jobin Yvon). The attenuated total reflectance surface enhanced infrared absorption spectroscopy (ATR-SEIRAS) experiments were performed with Perkin Elmer Spectrum 100 FT-IR spectrometer equipped with diamond attenuated total reflection (UATR) accessory, and a MCT detector that is cooled with liquid nitrogen.

**Electrochemical measurements.** Electrochemical measurements were performed in a three-electrode two-compartment cell separated by Nafion 117 membrane in an electrochemical workstation (CHI 660E). The catalyst (1 mg) was dispersed in mixed liquid of ethanol (75 μL), deionized water (19 μL) and Nafion solution (6 μL, 5 wt%), and then sonicated for 30 min to form a homogeneous ink. The mixture was then uniformly spread on carbon fiber paper of 1 × 1 cm^2^ to prepare the working electrode. Carbon rod and Ag/AgCl electrode (3.0 M KCl) were used as counter electrode and reference electrode, respectively. 0.5 M KHCO_3_ (pH ≈ 7.36) was used as electrolyte. All potentials were calibrated to the reversible hydrogen electrode (RHE) reference scale using E_RHE_ = E_Ag/AgCl_ + 0.059 × pH + 0.205. Linear sweep voltammetry (LSV) curves were performed with a scanning rate at 10 mV s^-1^ from -0.1 to -1.2 V (versus RHE) by using CO_2_ or Ar saturating the electrolyte. A pure CO_2_ gas (99.99%) was bubbled into aqueous electrolyte (40 mL) for 30 min prior to each experiment. The current was normalized by the geometric surface area. The electrolyte in the cathodic compartment was stirred at a rate of 200 rpm during electrolysis. During the experiments, CO_2_ gas was delivered at an average rate of 5 mL/min, and routed directly into the gas sampling loop of a gas chromatograph (shiweipx GC-2060). The gas chromatograph was equipped with a molecular sieve TDX-01 and Al_2_O_3_ capillary column with Ar (Ultra high purity) flowing as a carrier gas. The separated gas products were analyzed by a thermal conductivity detector (TCD) and a flame ionization detector (FID). The electrochemical surface area, ECSA = *R*_f_S, where S stands for the real surface area of the smooth metal electrode, which was generally equal to the geometric area of carbon fiber paper (in this work, S=1 cm^2^). The roughness factor *R*_f_ was estimated from the ratio of double-layer capacitance *C*_dl_ for the working electrode and the corresponding smooth metal electrode (Specific capacitance for carbon was reported as 40 μF cm^-2^ [1]). Therefore, *R*_f_ = *C*_dl_/40 μF cm^-2^. The *C*_dl_ was determined by measuring the capacitive current associated with double-layer charging from the scan-rate dependence of cyclic voltammetric stripping. The potential window of cyclic voltammetric stripping was 0.1 V to 0 V versus RHE. The scan rates included 5 mV/s, 10 mV/s, 20 mV/s, 40mV/s, and 60 mV/s. The *C*_dl_ was estimated by plotting the ∆j = (*j*_a_ − *j*_c_) at 0.50 V (where *j*_c_ and *j*_a_ are the cathodic and anodic current densities, respectively) versus RHE against the scan rates, in which the slope was twice that of *C*_dl_.

For gaseous products, the Faradaic efficiency (FE*_gas_*) was calculated as follows:

$\mathrm{FE}_{gas}=\frac{q_{g}}{q_{tot}}\times100\%=\frac{v\times V\times N\times F}{60\times24000\times j}\times100\%$ (1)

Where *v* was the CO_2_ flow rate, *V* was the measured product concentration in the GC sample loop, *N* = 2 was the number of electron transfer to form a molecule of CO or H_2_, *F* was the Faraday constant (96485 C mol^-1^), and *j* was the total current.

For liquid products, the following method was used for the calculation of Faradaic efficiency (FE*_liquid_*):

$\mathrm{FE}_{liquid}=\frac{q_{l}}{q_{tot}}\times100\%=\frac{F\times c_{l}\times V_{cell}\times n_{l}}{q_{tot}}\times100\%$ (2)

Where *c*_l_ (mol L^-1^) was the concentration of formate that was calculated from ^1^H NMR spectroscopy, *F* was Faraday constant, *V*_cell_ (L) was the electrolyte volume in the cell, and *q*_tot_ (C) was the passed total charge.

***Operando* XRD.** *Operando* XRD experiments were performed using an X-Ray Polycrystalline Diffractometer (Bruker AXS D8 Advance). The 2.2 kW monochromatic x-ray beam was focused on the ceramic X-ray tube of the Cu target. The goniometer is placed vertically. The XRD patterns were collected with a Links energy dispersion array detector. A self-made electrolytic cell was used for electrochemical experiments. The carbon paper, Ag/AgCl (3 M KCl) and Pt foil were used as working electrode, reference electrode and counter electrode, respectively. The measurements were performed in CO_2_-saturated 0.5 M KHCO_3_ electrolyte with grazing incidence geometry. The sample was first tested at OCP, and then the potentials were gradually increased. The resting time at each potential before the measurement was 1 min.

***Operando* Raman spectroscopy.** Raman spectroscopic measurements were performed using a LabRAM HR800 confocal microscope (Horiba Jobin Yvon). A large working distance objective lens (50 times magnification, 0.37 mm focal length) was applied with a numerical aperture of 0.75 in order to focus a diode-pumped solid-state laser beam (excitation wavelength of 632 nm, power of 3 mW) on the sample. The Raman signal were collected in backscattering geometry using a home-made electrochemical cell. In the above systems, platinum wire and an Ag/AgCl electrode were used as the counter and reference electrode, respectively.

***Operando* XAFS.** *Operando* XAFS measurements were performed in Shanghai Synchrotron Radiation Facility (BL14W1, SSRF). A home-made cell was designed. For *operando* measurements, the cell was filled with the electrolyte (0.5 M KHCO_3_). Ag/AgCl and carbon rod were used as the reference and counter electrode, respectively. The carbon-fiber electrode with catalyst loading of 1 mg cm^-2^ was served as the working electrode. *Operando* XAFS were recorded at a given current density. In the *operando* XAFS measurements, in keeping with electrochemical test, the initial testing time is after electrochemical treatment for about 30 minutes at corresponding cathodic reduction current. Pipes were connected to the peristaltic pump to cycle the electrolyte, and to eliminate the effect of bubble as much as possible. The X-ray was monochromatized by a double-crystal Si(111) monochromator.

***Operando* FTIR.** The attenuated total reflectance surface enhanced infrared absorption spectroscopy (ATR-SEIRAS) experiments were performed with Perkin Elmer Spectrum 100 FT-IR spectrometer equipped with diamond attenuated total reflection (UATR) accessory, and a MCT detector that was cooled with liquid nitrogen. The spectral resolution was set to 4 cm^-1^, and 76 interferograms were co-added for each spectrum. The spectra were given in transmittance units defined as T = (*R*-*R_0_*)/*R_0_*, where *R* and *R_0_* represent the reflected IR intensities, corresponding to the sample and reference single beam spectrum, respectively. A Si face-angled crystal with incident angle of 60° was used as reflection element. The ultrathin Au foil was deposited for IR-signal enhancement and conduction of electrons. The electrocatalyst was dropped onto Au film to serve as working electrode for SEIRAS experiments with the loading of 1 mg cm^-2^. A platinum wire and an Ag/AgCl electrode were used as counter and reference electrode in all tests, respectively. The CO_2_-saturated 0.5 M KHCO_3_ was used as electrolyte. Chronopotentiometry method was used in this experiment at different potentials (-0.1 to -1.2 V vs. RHE without *iR*-correction). The SEIRAS spectra were collected during the chronopotentiometry test.

**DFT calculations.** DFT calculations were performed using the Vienna ab-initio simulation package (VASP) [2, 3]. A plane-wave basis set with a cut-off energy of 450 eV was used to expand the Kohn-Sham wave functions. The interactions between the ionic cores and valence electrons were described using a projector augmented wave (PAW) method [4, 5], and the exchange-correlation effects were self-consistently described within the Perdew-Burke-Ernzerh of generalized gradient approximation (GGA-PBE) [6, 7]. The occupation of electronic states was determined using Gaussian smearing method [8] and the smearing width was 0.01 eV. The DFT+U formalism was used to describe the localized (strongly correlated) 4f electrons in cerium, as implemented by Dudarev [9]. For Ce, U_Ce_ – J_Ce_ = 4.5 eV was used [10].

Considering that the CO_2_RR was carried out at aqueous phase condition, a fully hydroxylated ceria cluster (Ce_3_O_7_H_7_) supported on Bi(110) surface is adopted to simulate the D-CeO_x_/Bi catalyst, denoted as Ce_3_O_7_H_7_/Bi(110) [10]. Bi(110) surface was adopted to model the Bi catalyst for reference. Bi(110) substrate was modeled as a four-layered slab with (3×3) unit cells, of which the bottom two layers were fixed. The top two layers of Bi(110), the CeO_7_H_7_ cluster and the surface species were fully relaxed. A vacuum spacing of 12 Å along the normal direction (z) to the surface and a 1×2×1 Gamma centered k-point sampling were used for the models. Optimized geometries were obtained by minimizing the maximum force on any ion until it is less than 0.05 eV/Å.

The computational hydrogen electrode (CHE) model was used to determine the reaction energies of electrochemical reaction as a function of potential U vs. RHE [11, 12]. The Gibbs free energies were calculated using the following equation:

$G(\mathrm{T})=E_{DFT}+ZPE+\int_{0}^{T} C_{V,\mathrm{vib}}dT-T\cdot S(\mathrm{T})$ (3)

where *E_DFT_*, *ZPE*, *C_V,vib_*, *T* and *S(T)* are the total energy from DFT calculation, the zero point energy (ZPE) correction, heat capacity, the reaction temperature (298 K) and entropy, respectively. For the surface species, *ZPE*, *C_V,vib_* and *S(T)* are calculated using equations (4-6):

$ZPE=\sum_{i}^{\#vibs} (\frac{1}{2})h\upsilon_{i}$ (4)

$\int_{0}^{T} C_{V,vib}dT=\sum_{i}^{\#vibs} \frac{hv_{i}}{e^{\frac{hv_{i}}{k_{B}T}}-1}$ (5)

$S(\mathrm{T})=k_{B}\sum_{i}^{\#vibs} [\frac{h\upsilon_{i}}{k_{B}T(\mathrm{e}^{\frac{h\upsilon_{i}}{k_{B}T}}-1)}-\ln(1-\mathrm{e}^{\frac{-h\upsilon_{i}}{k_{B}T}})]$ (6)

where *k_B_*, h and $v_{i}$ are Boltzmann factor, Planck constant and the calculated vibrational frequencies, respectively. Gas-phase molecules were treated as ideal gas with corrections. We applied in this work the same free energy corrections for the gaseous species and solvation effect as in Refs. [13, 14].


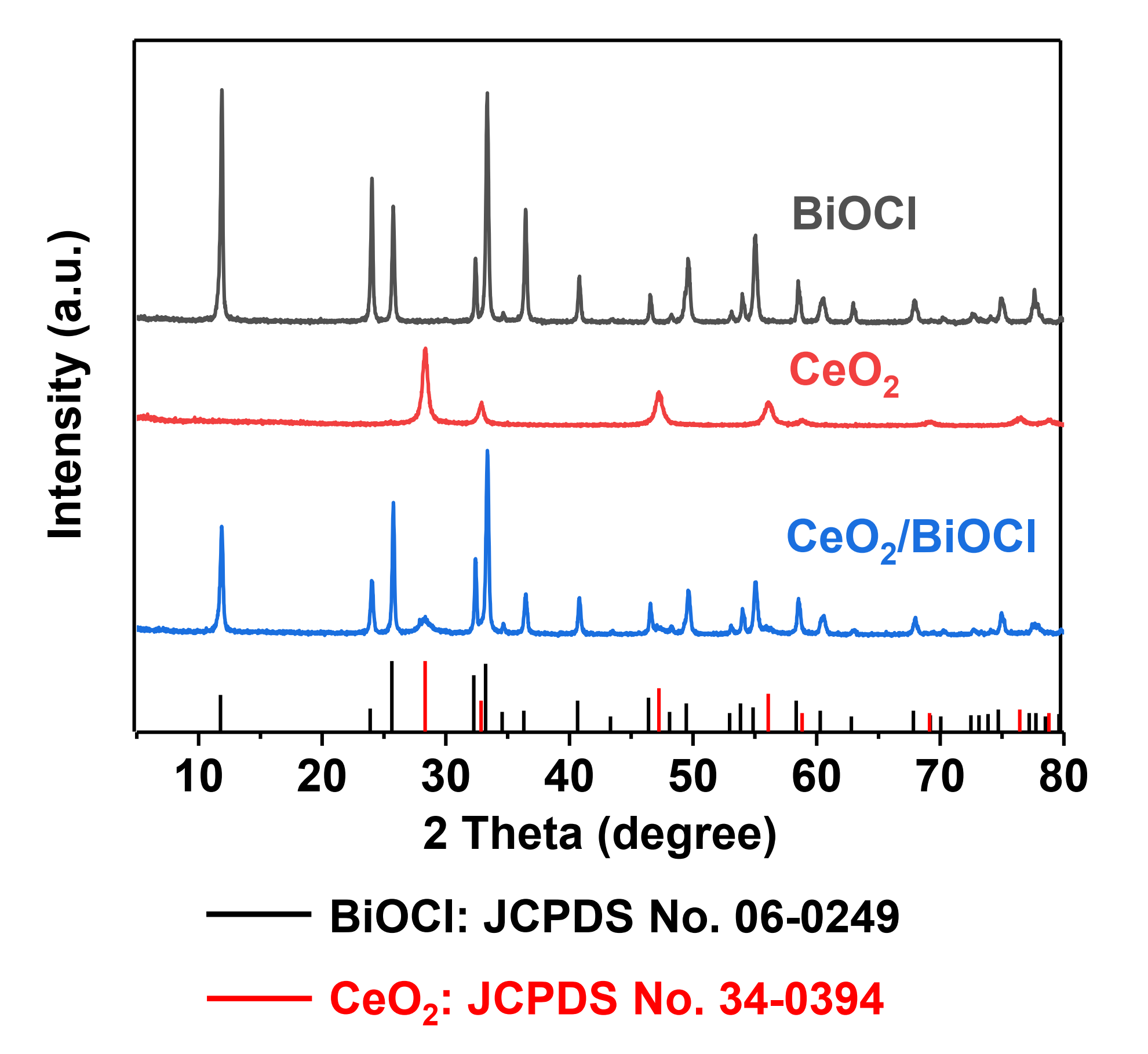


**Supplementary Figure 1**. XRD patterns of BiOCl, CeO_2_ and CeO_2_/BiOCl.


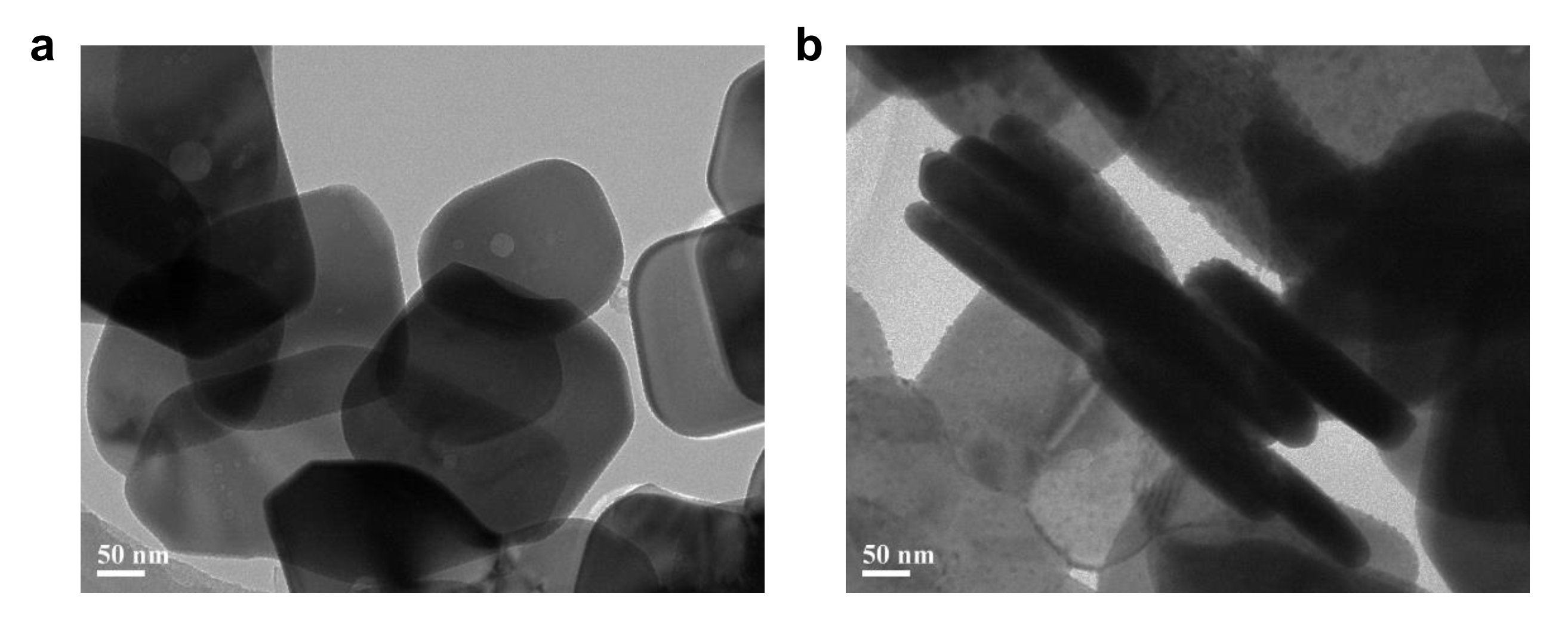


**Supplementary Figure 2**. TEM images of BiOCl.


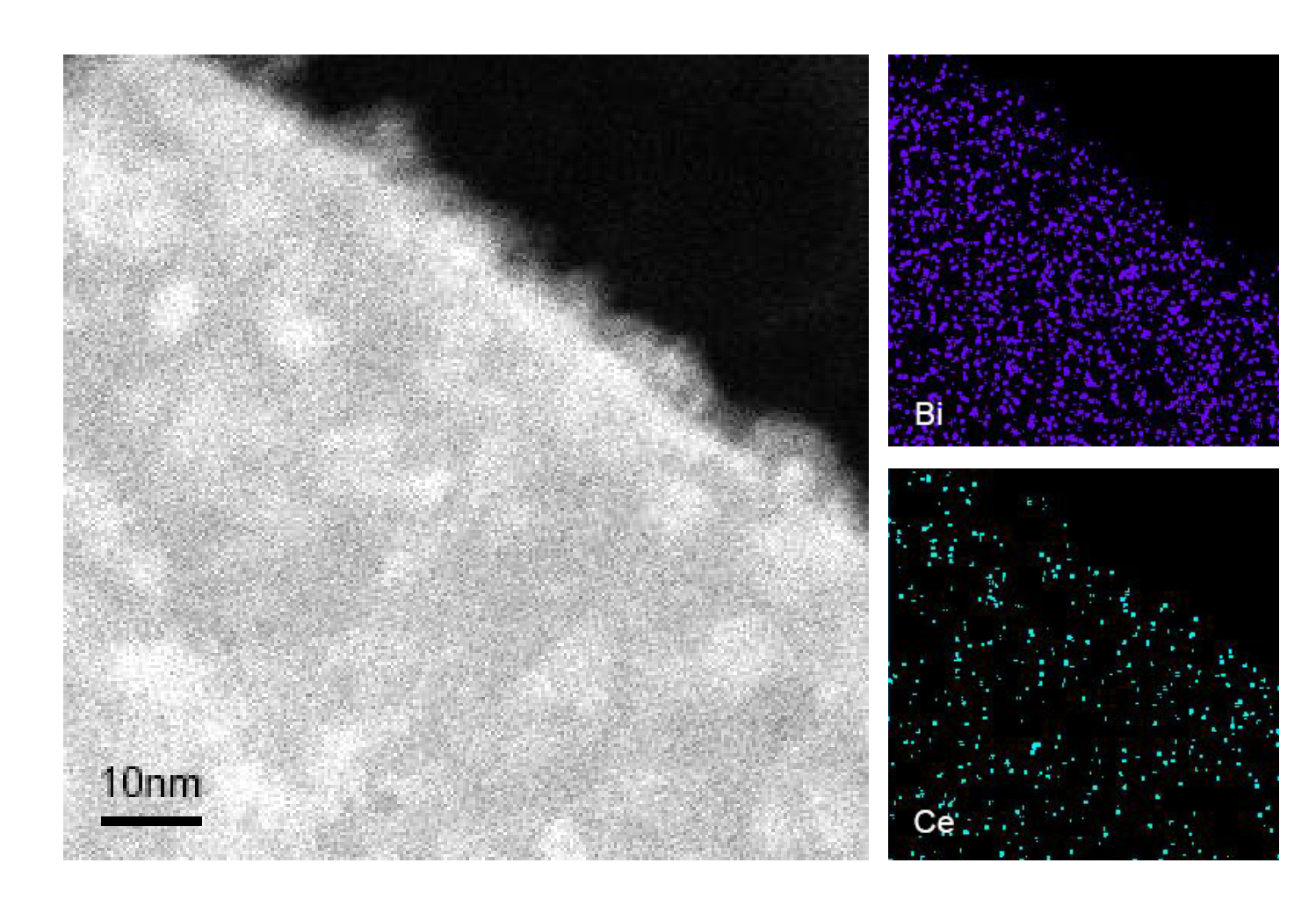


**Supplementary Figure 3**. EDX elemental maps of CeO_2_/BiOCl.


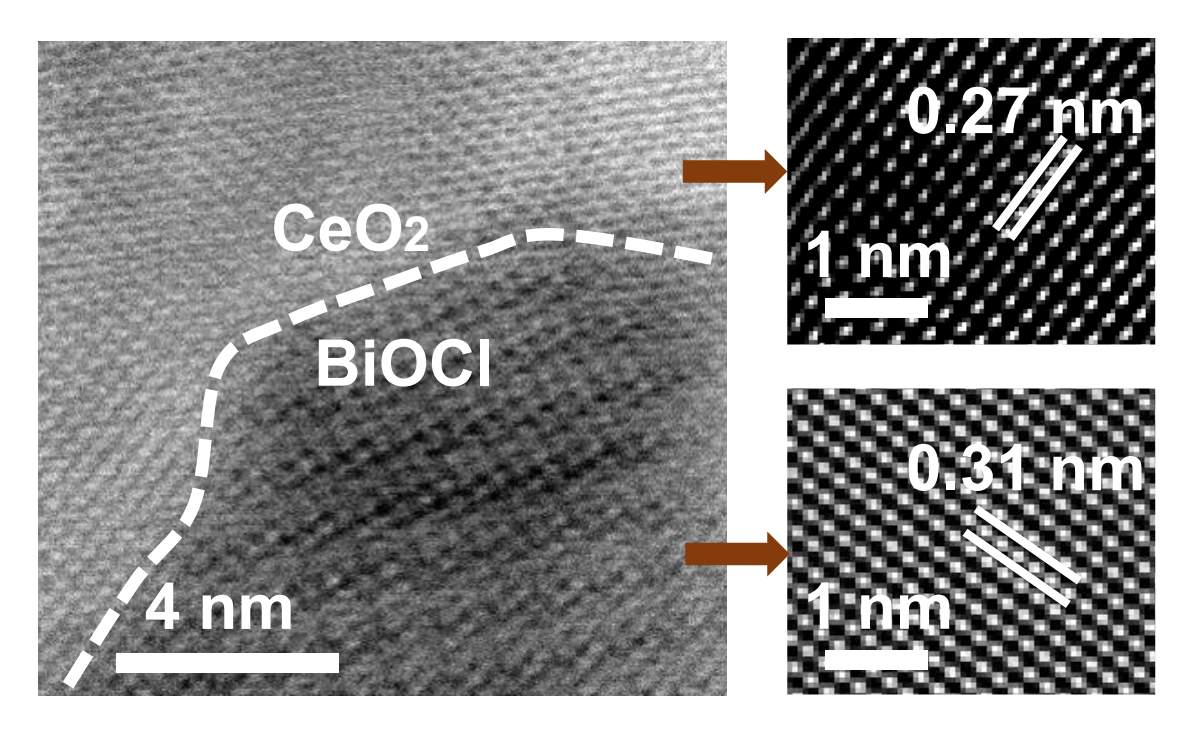


**Supplementary Figure 4.** TEM and corresponding fast Fourier Transform images of the CeO_2_/BiOCl.


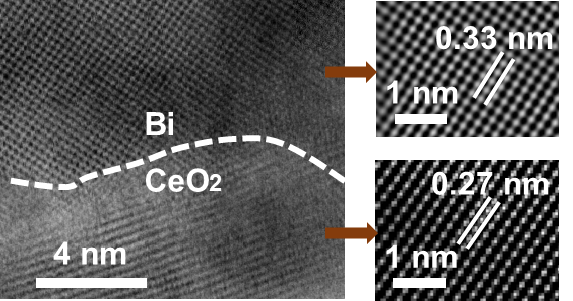


**Supplementary Figure 5.** TEM and corresponding fast Fourier Transform images of D-CeO_x_/Bi.


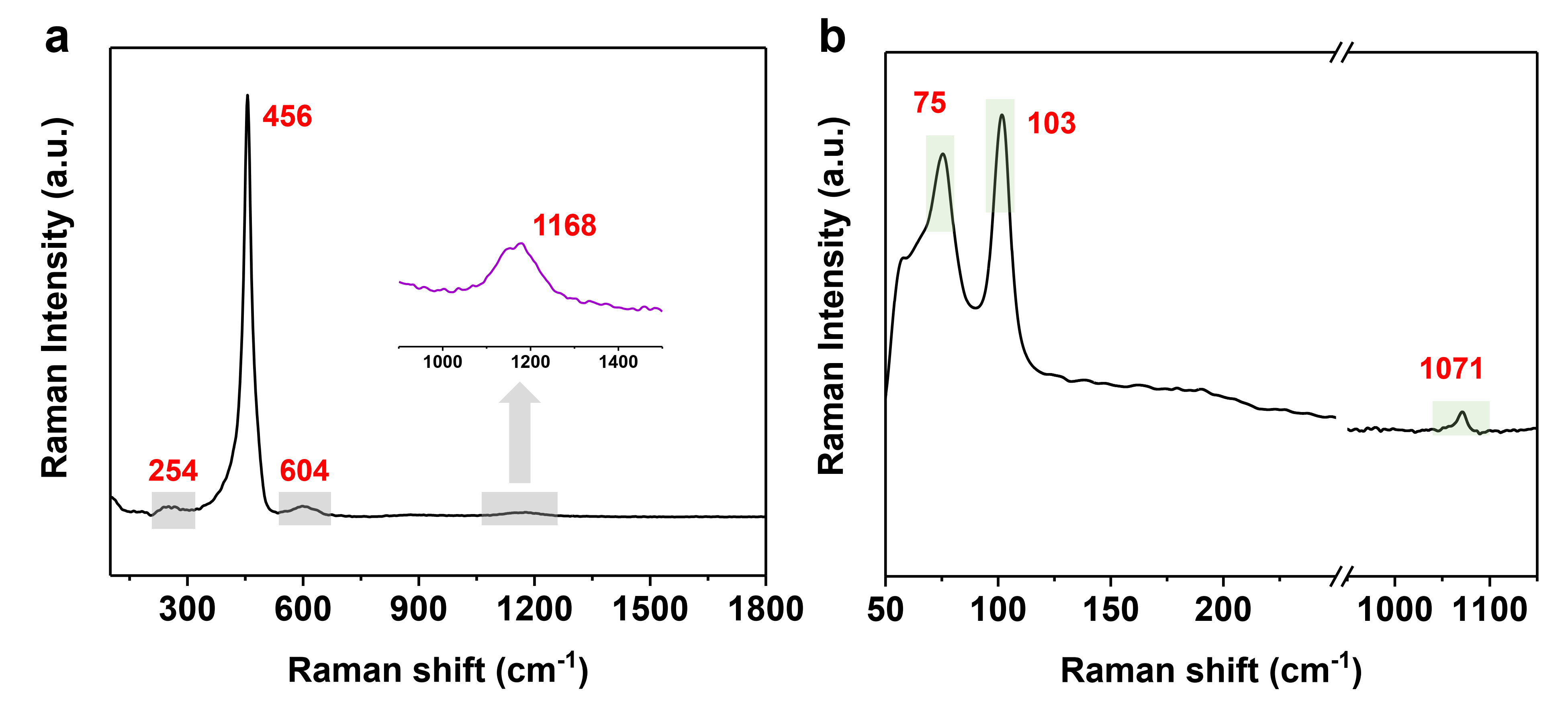


**Supplementary Figure 6.** Ex-situ Raman spectra of the (a) D-CeO_x_ and (b) D-CeO_x_/Bi.


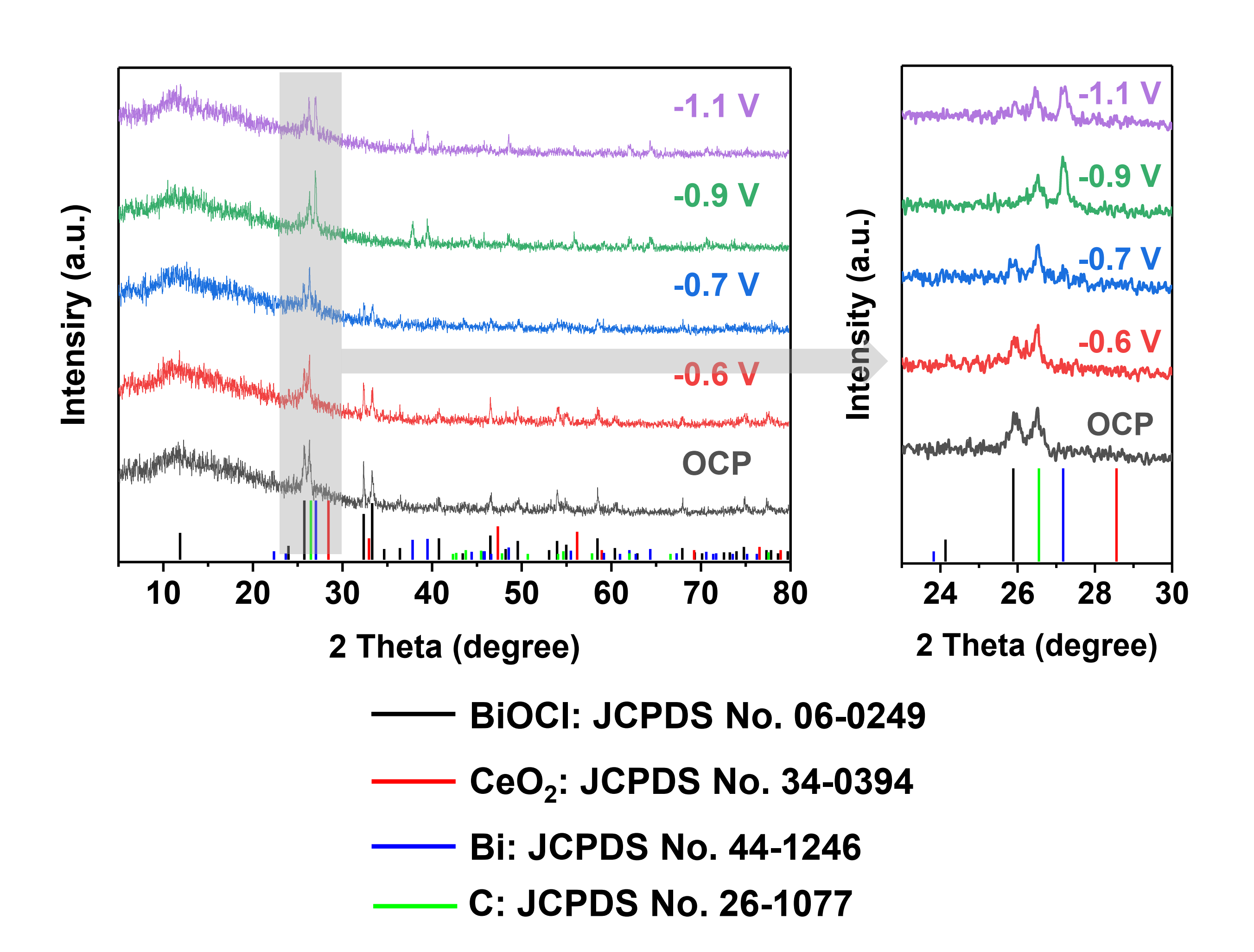


**Supplementary Figure 7.** *Operando* XRD patterns of CeO_2_/BiOCl loaded carbon paper.


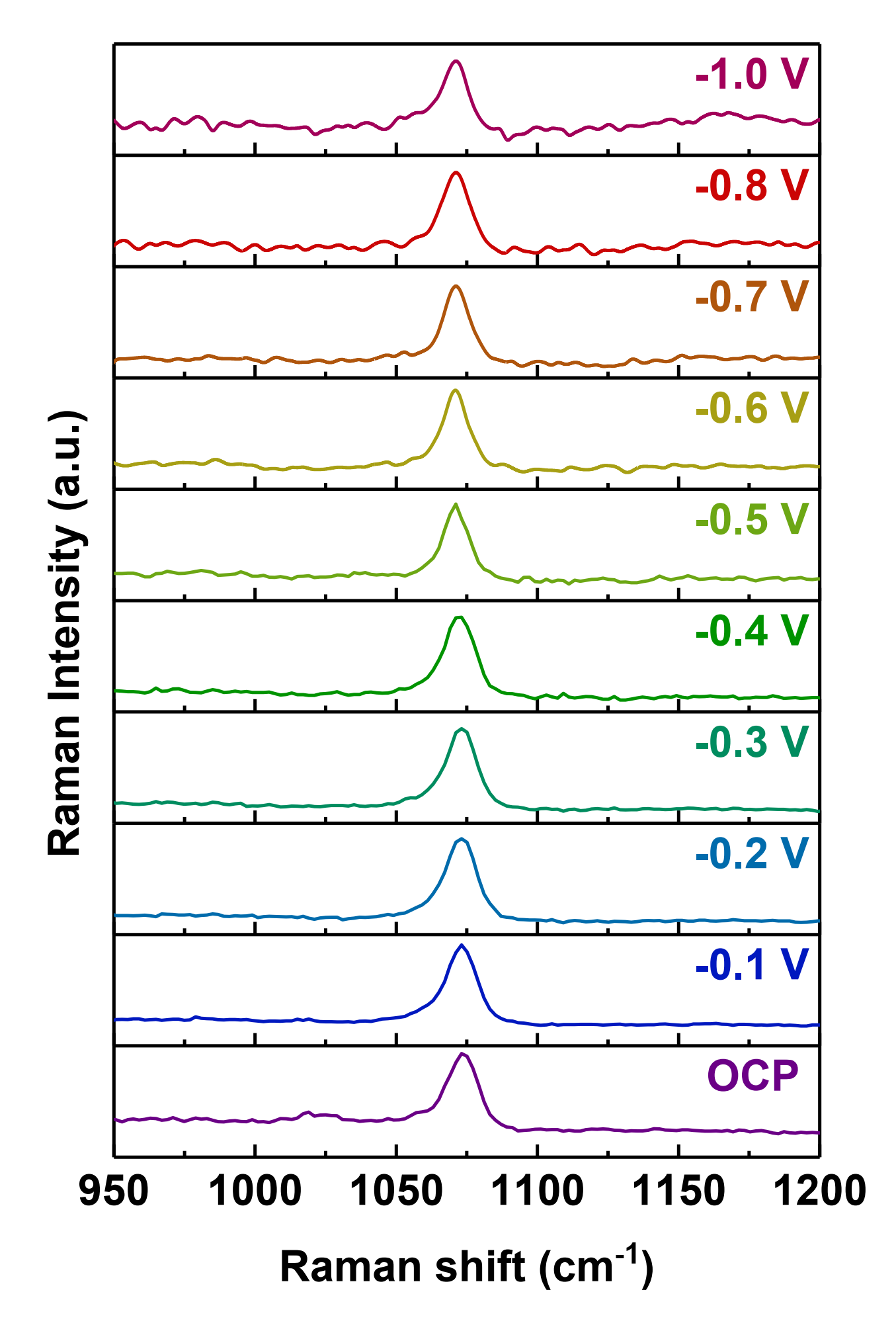


**Supplementary Figure 8.** Potential-dependent *operando* Raman spectra of the CeO_2_/BiOCl at wavenumber region of 950 to 1200 cm^-1^.


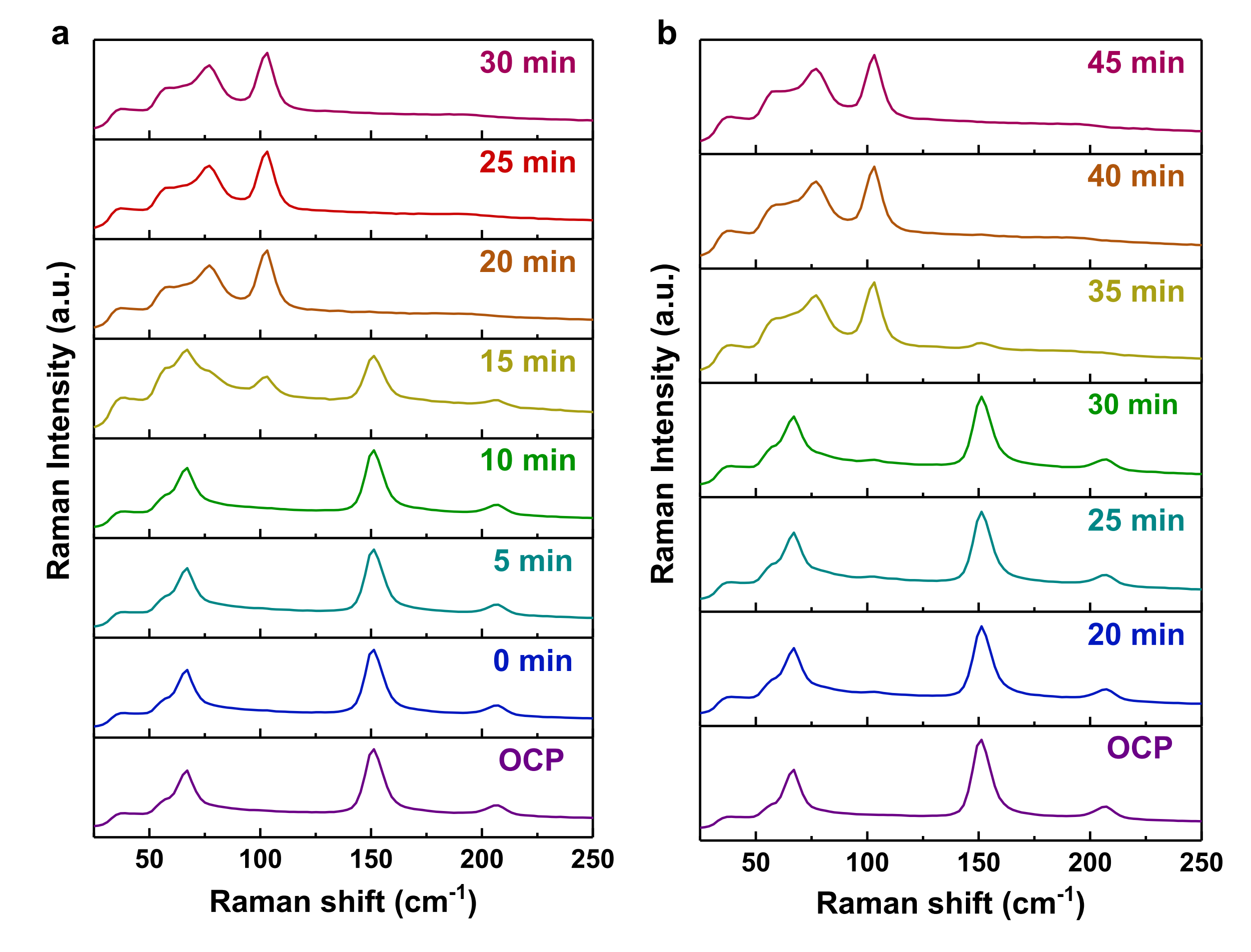


**Supplementary Figure 9.** Time-dependent *operando* Raman spectra of the (a) CeO_2_/BiOCl and (b) BiOCl at -0.9 V versus RHE.


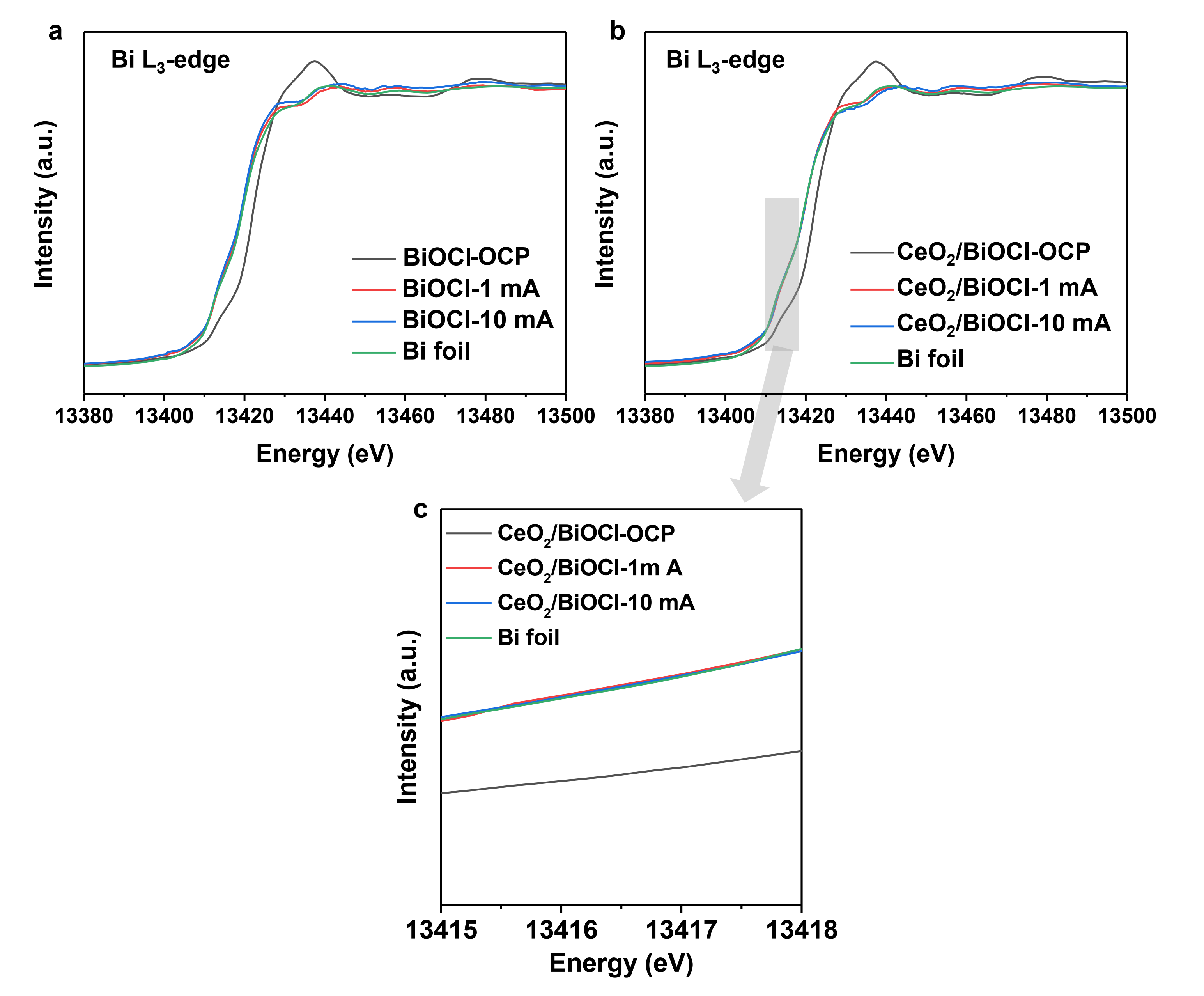


**Supplementary Figure 10.** *Operando* X-ray absorption near edge structure spectra of the (a) BiOCl, (b) CeO_2_/BiOCl and (c) local enlargement at the Bi L_3_-edge with cathodic reduction current of 1 and 10 mA/cm^2^.


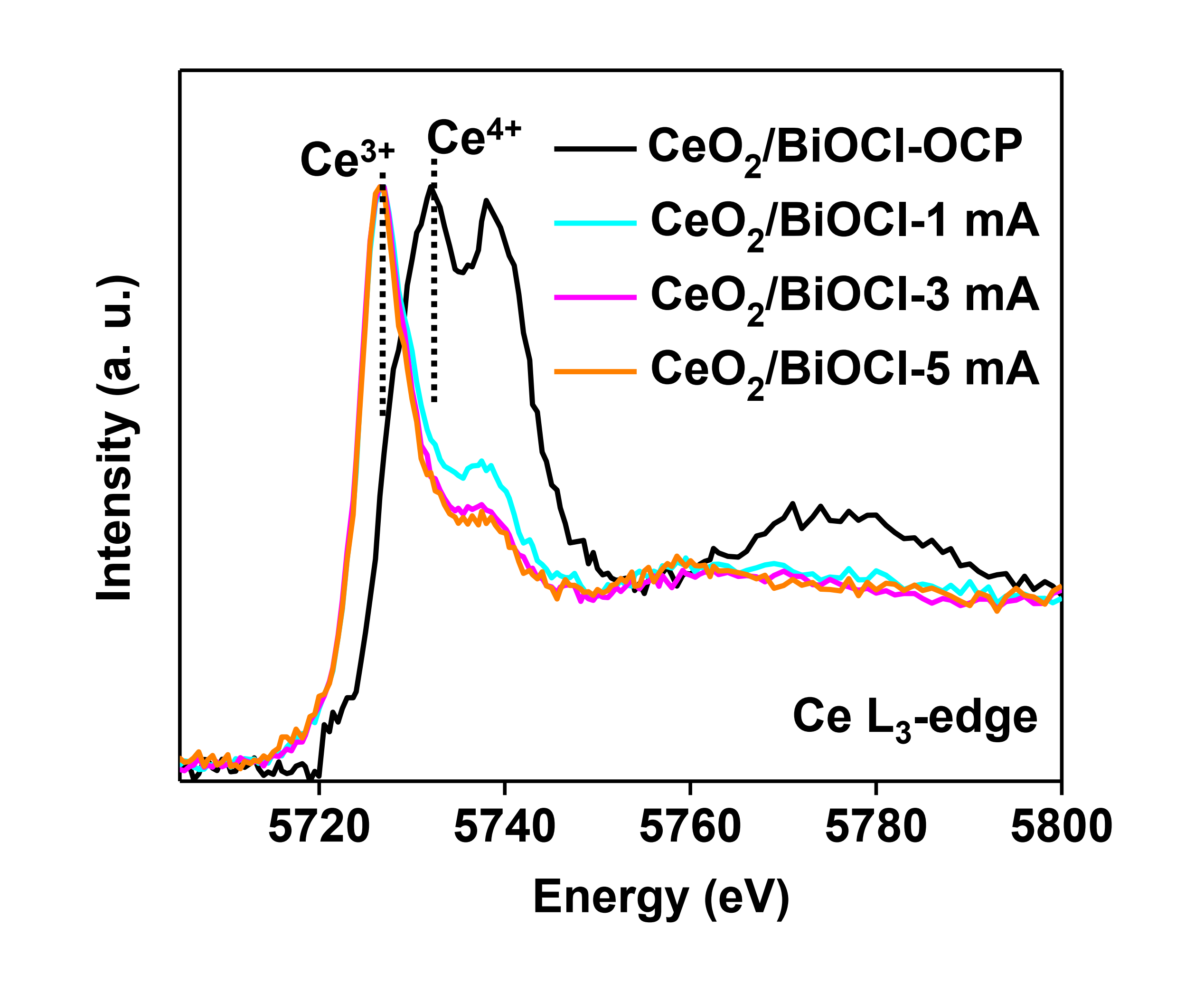


**Supplementary Figure 11.** *Operando* X-ray absorption near edge structure spectra of the CeO_2_/BiOCl and at the Ce L_3_-edge with cathodic reduction current of 1, 3 and 5 mA/cm^2^.


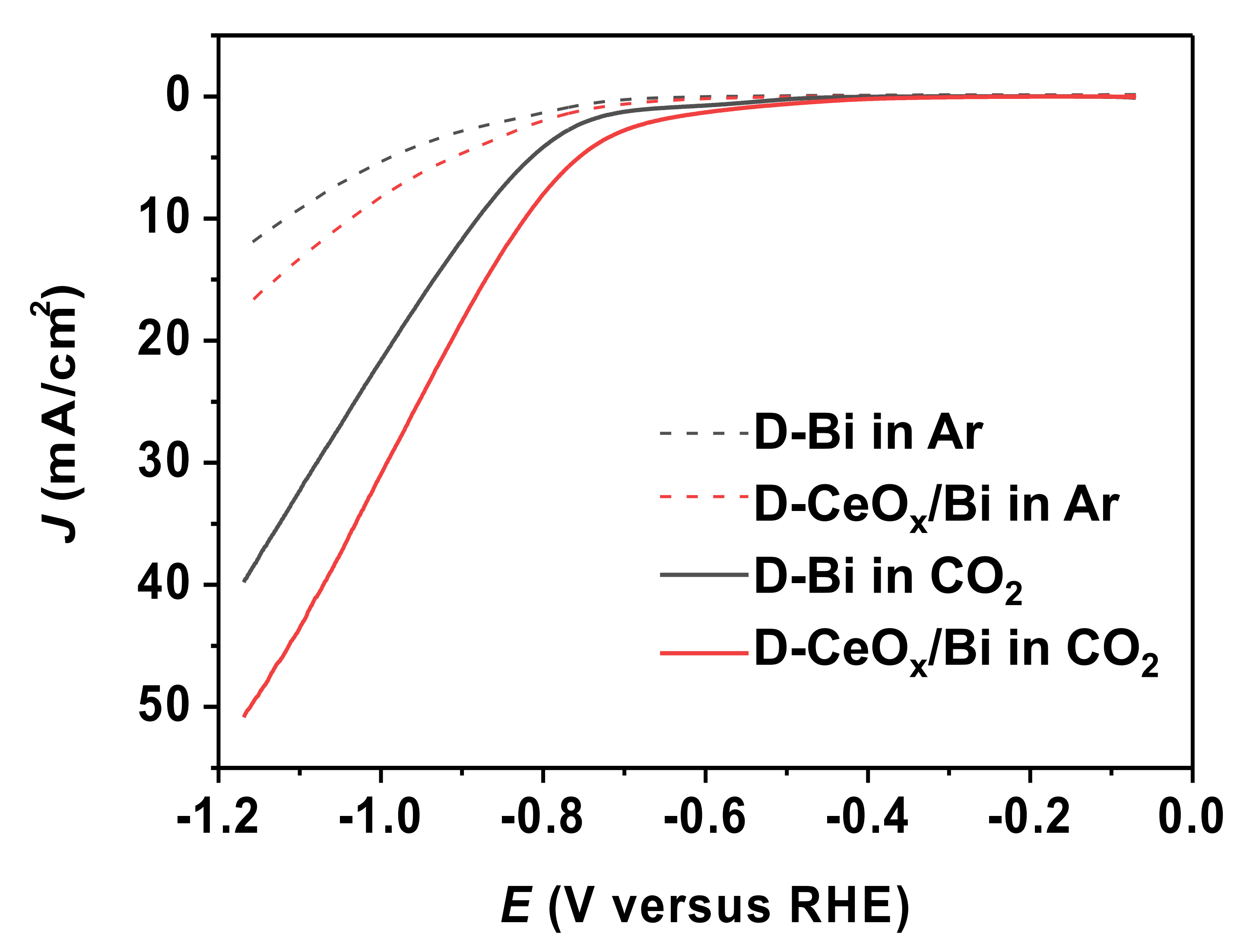


**Supplementary Figure 12.** LSV curves of the D-Bi and D-CeO_x_/Bi in the Ar- and CO_2_-saturated electrolytes.


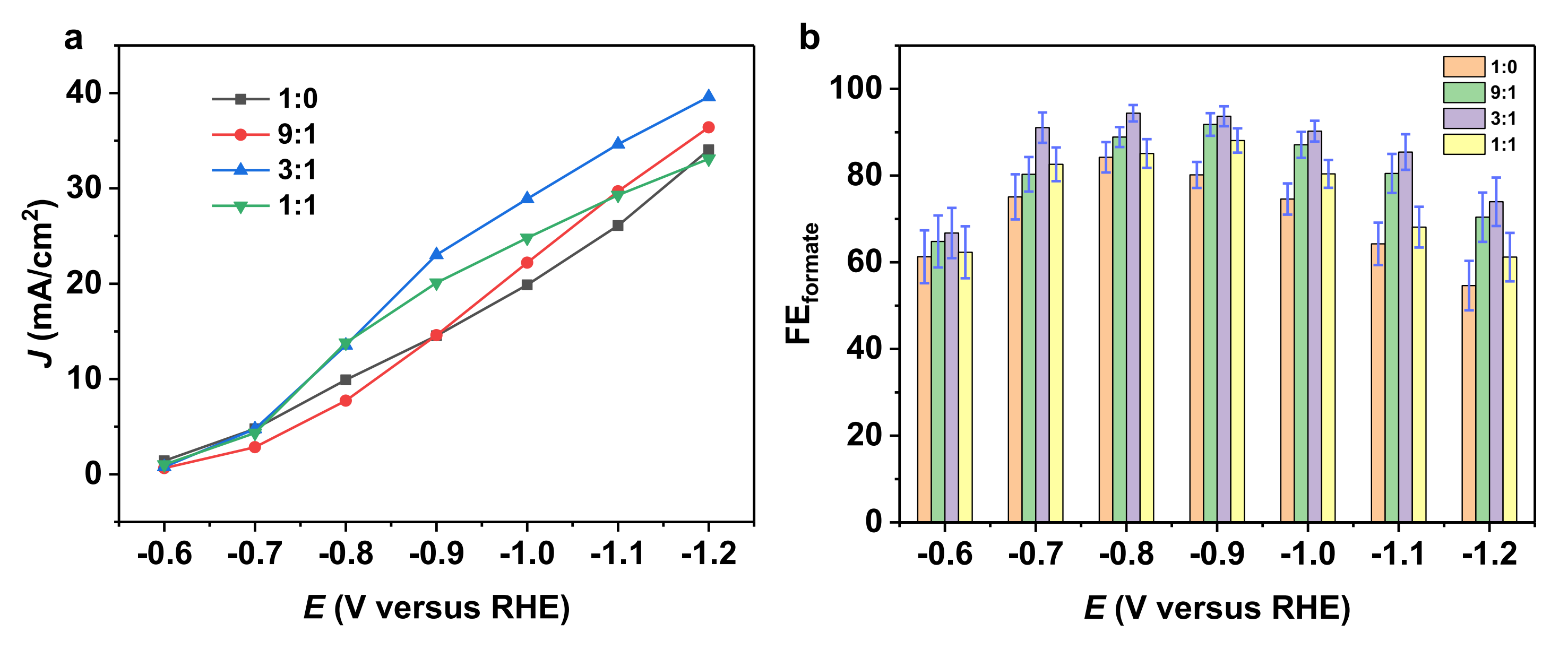


**Supplementary Figure 13.** (a) Current densities and (b) FE_formate_ of the D-CeO_x_/Bi with different Bi/Ce ratios.


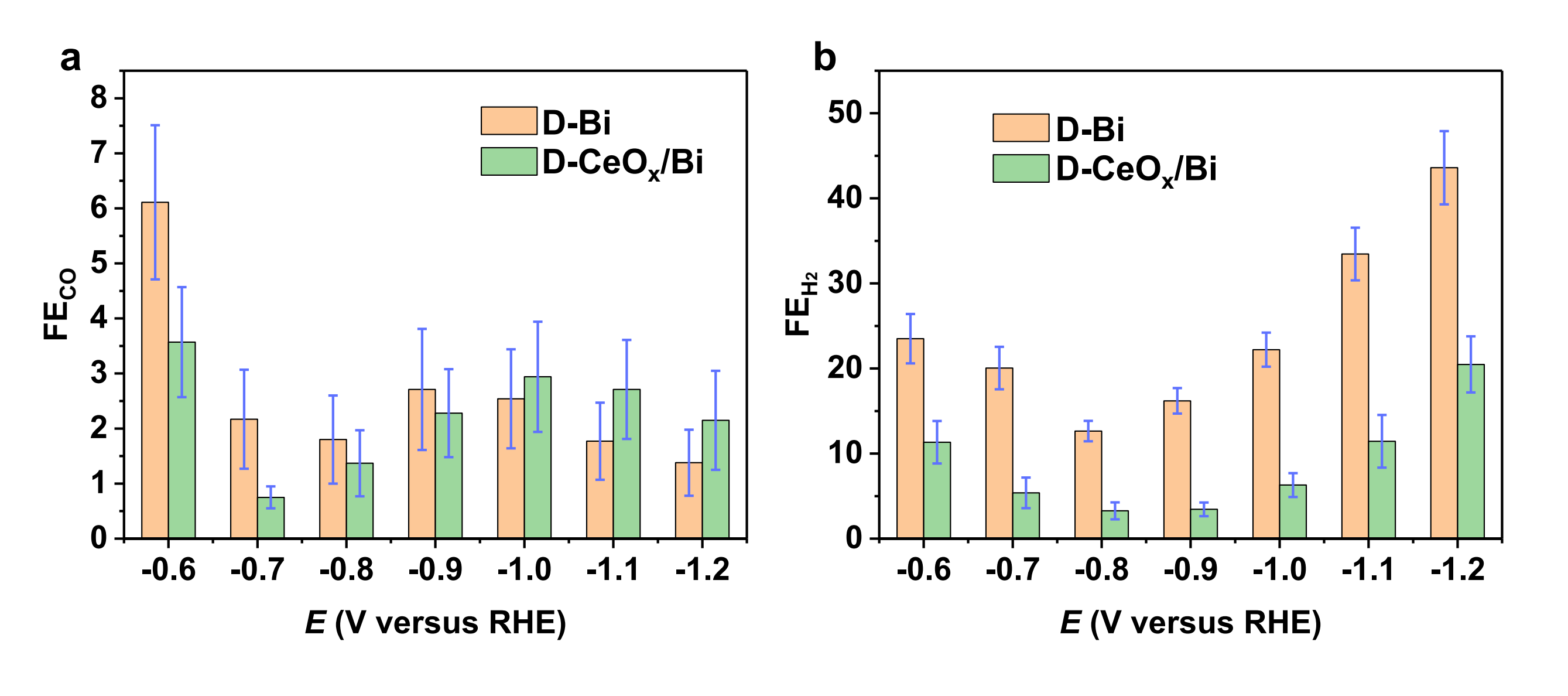


**Supplementary Figure 14.** (a) FE_CO_ and (b) FE_H2_ of the D-Bi and D-CeO_x_/Bi.


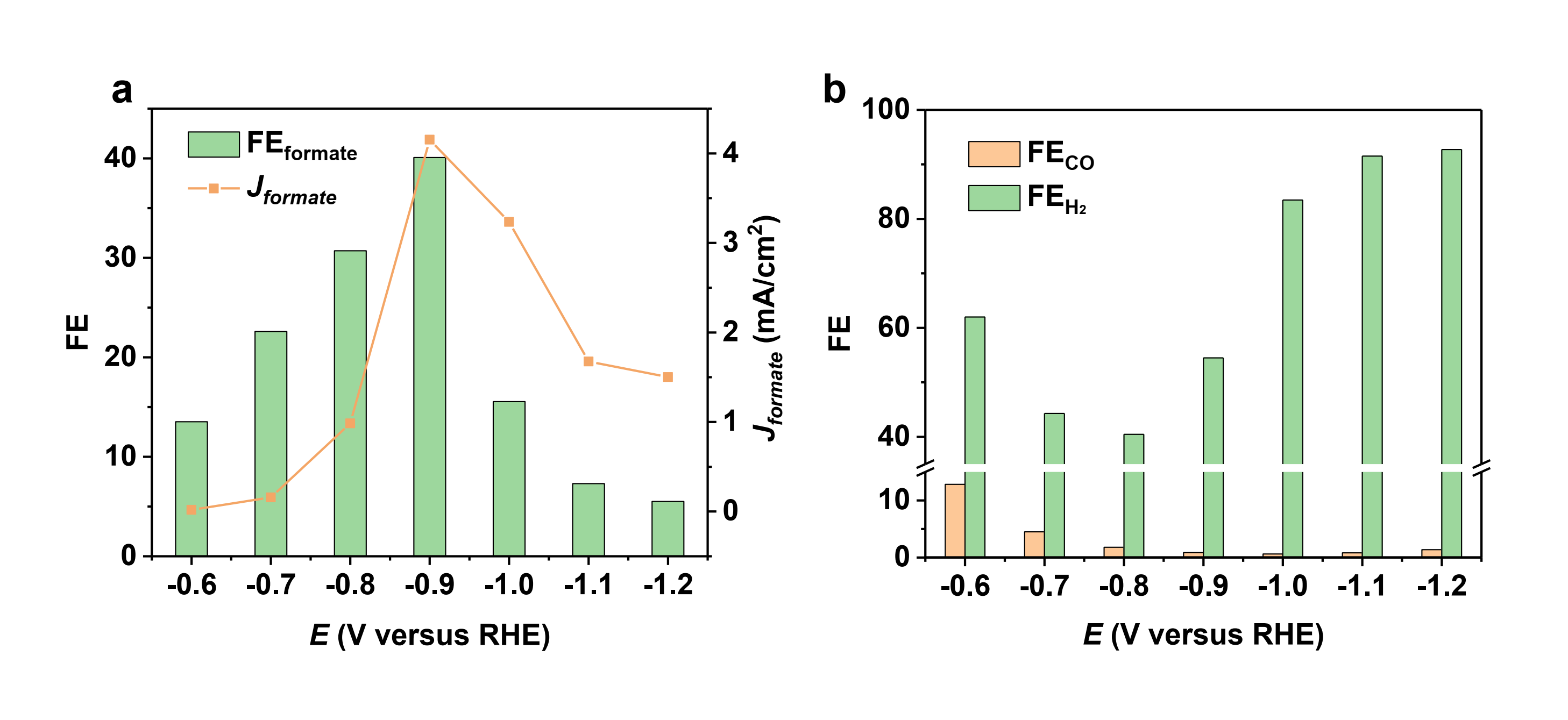


**Supplementary Figure 15.** (a) FE_formate_ and formate partial current density, (b) FE_H2_ and FE_CO_ of the D-CeO_x_.


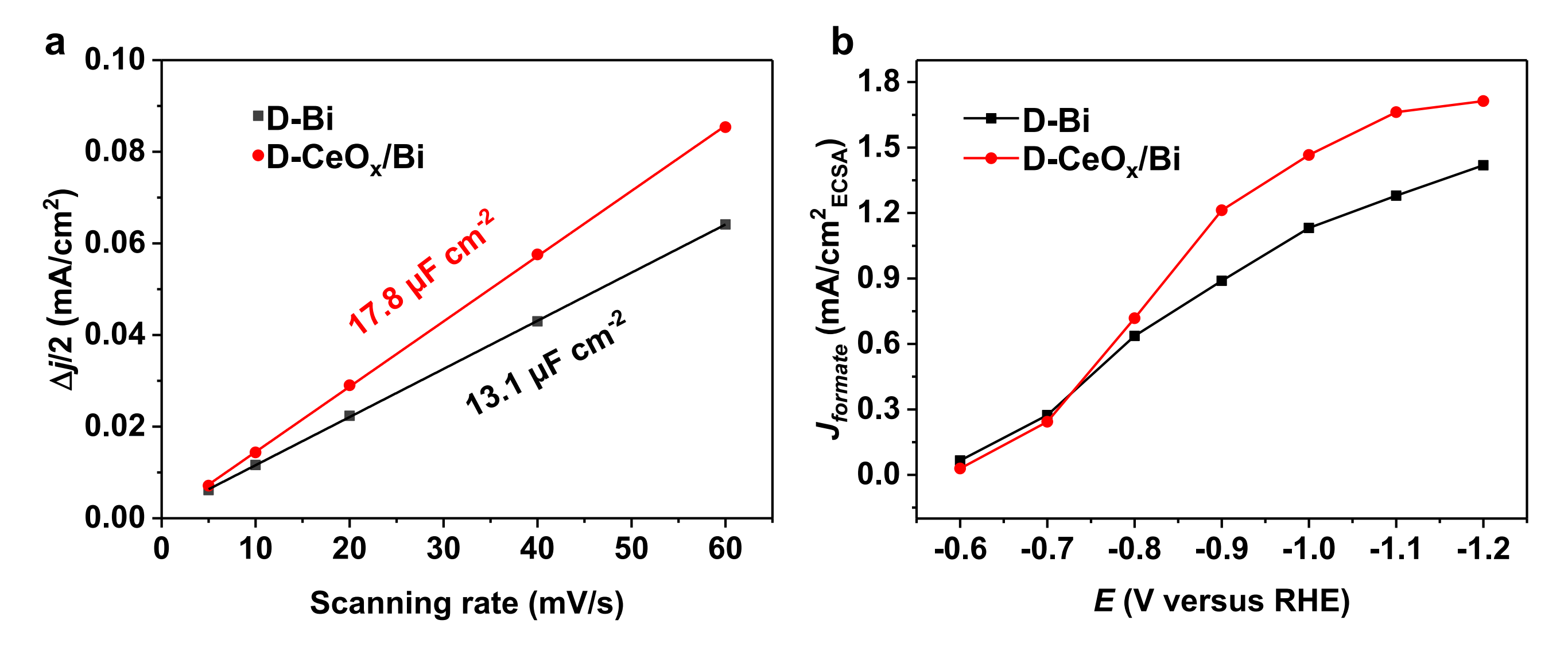


**Supplementary Figure 16.** (a) ECSA and (b) ECSA-normalized formate partial current density of the D-Bi and D-CeO_x_/Bi.


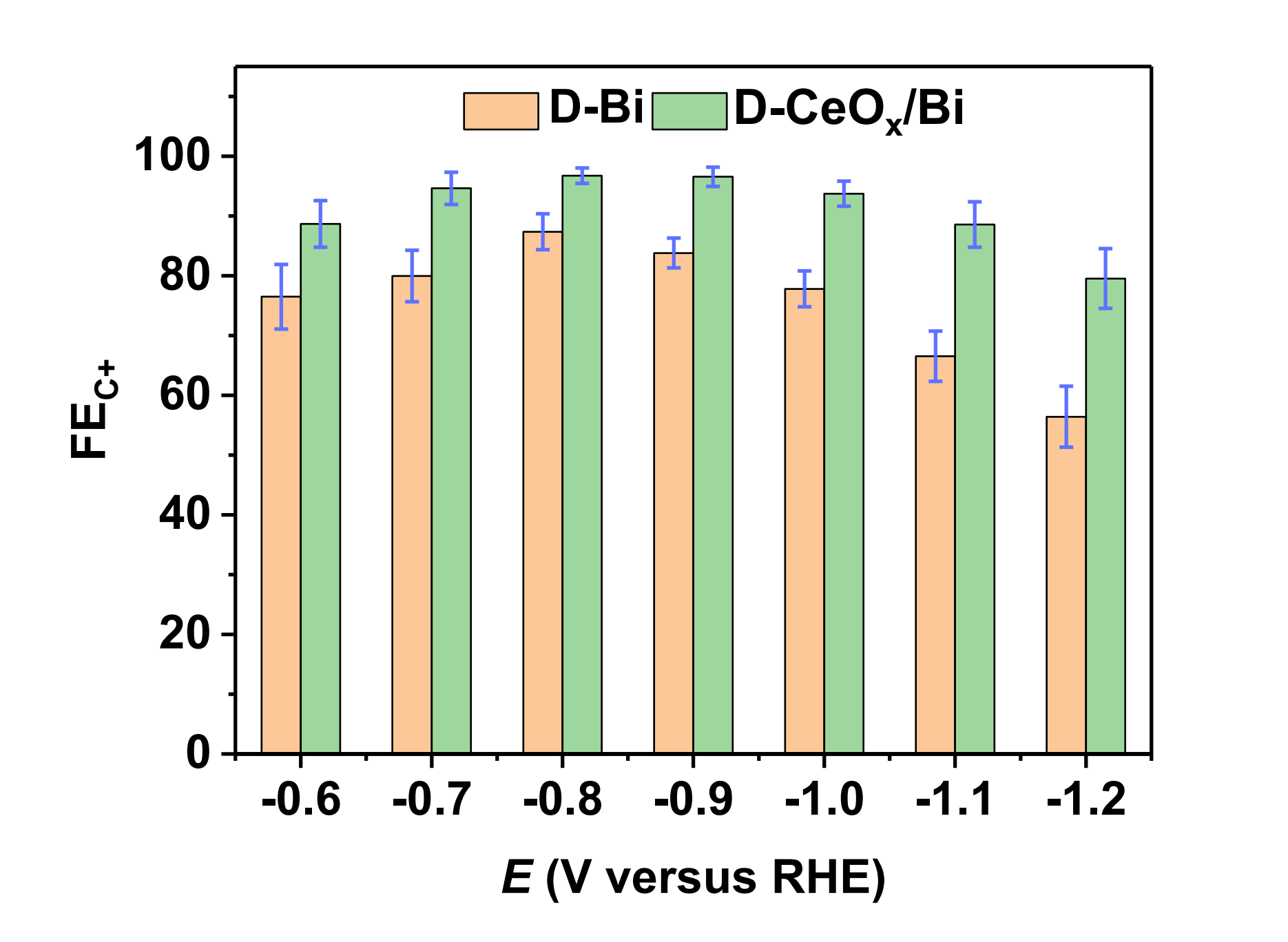


**Supplementary Figure 17.** FE_C+_ of products that contain carbon element (formate and CO) of the D-Bi and D-CeO_x_/Bi.


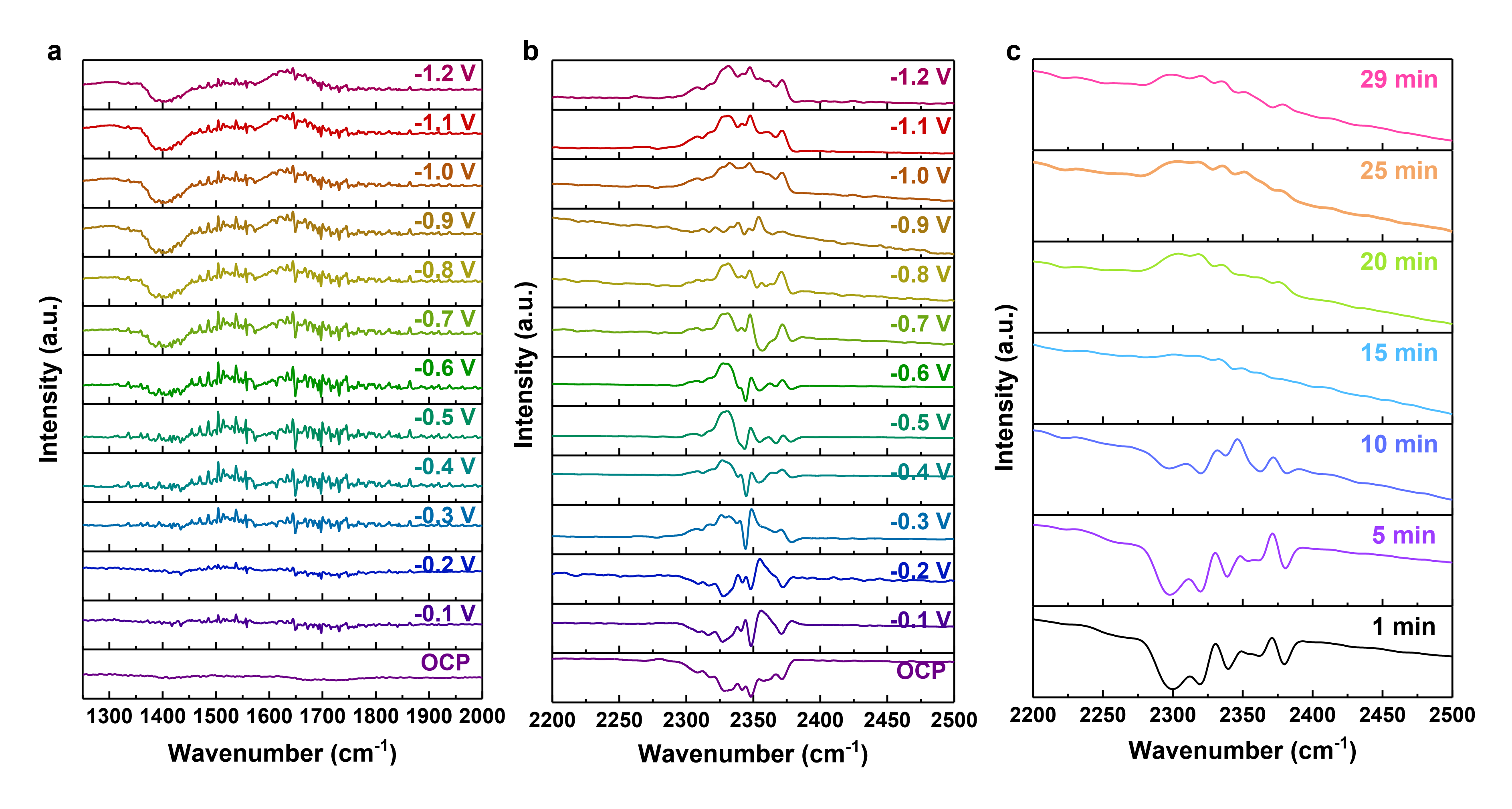


**Supplementary Figure 18.** Potential-dependent *operando* FTIR spectra of the D-CeO_x_/Bi at (a) 1250 to 200 cm^-1^ and (b) 2200 to 2500 cm^-1^ as well as (c) time-dependent *Operando* FTIR spectra of the D-CeO_x_/Bi at -0.9 V versus RHE.


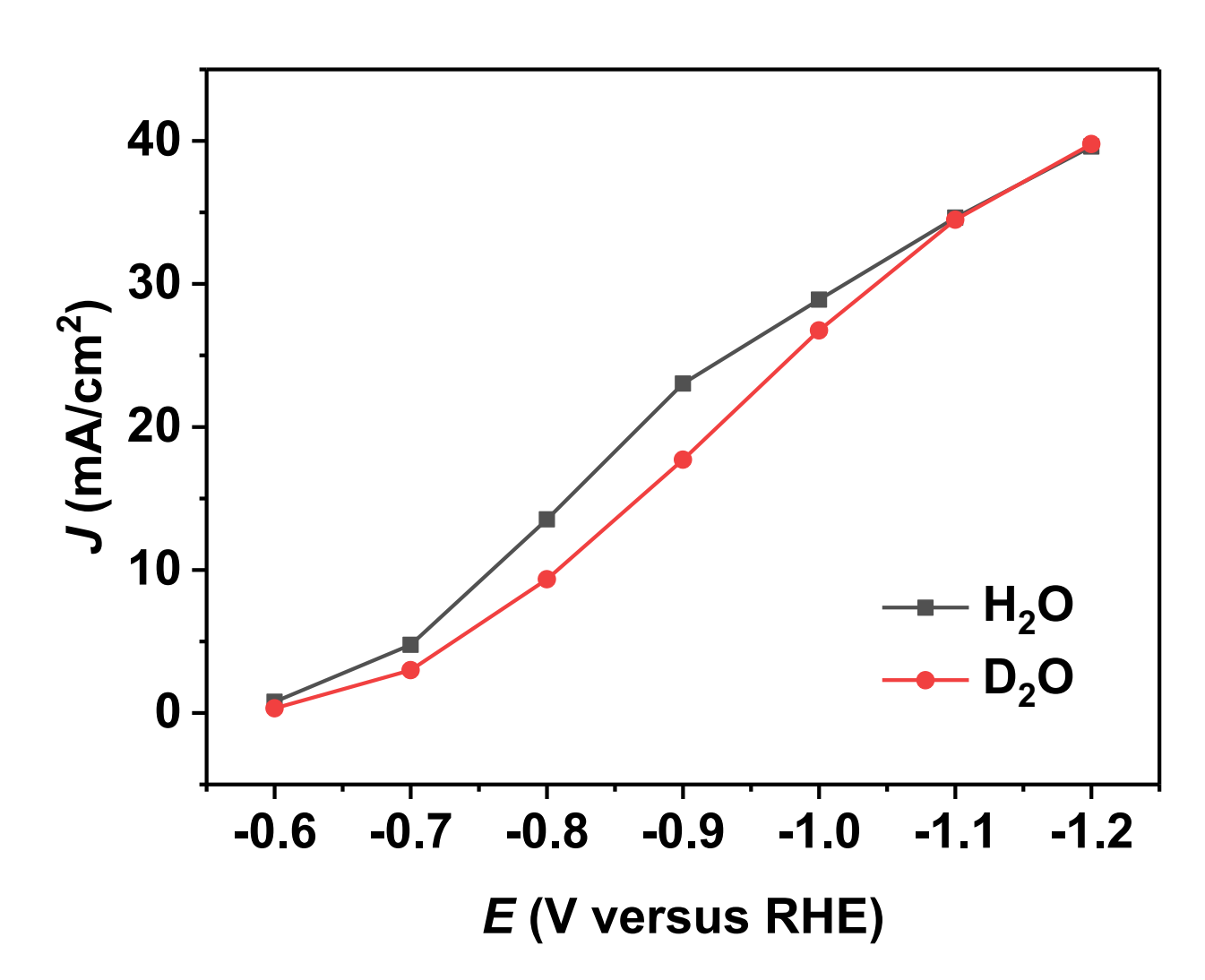


**Supplementary Figure 19.** Potential-dependent current densities of the D-CeO_x_/Bi under H_2_O and D_2_O conditions.


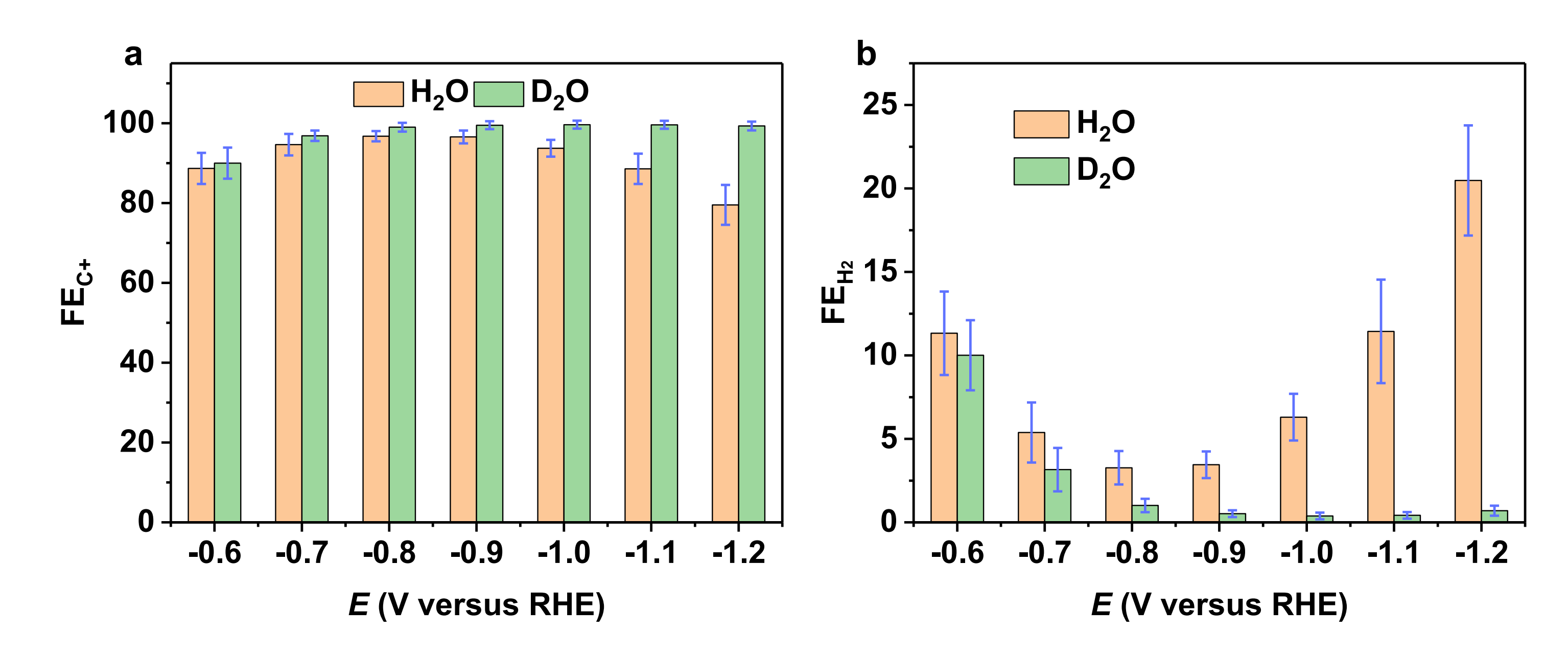


**Supplementary Figure 20.** (a) FE_C+_ and (b) FE_H2_ of the D-CeO_x_/Bi under H_2_O and D_2_O conditions.


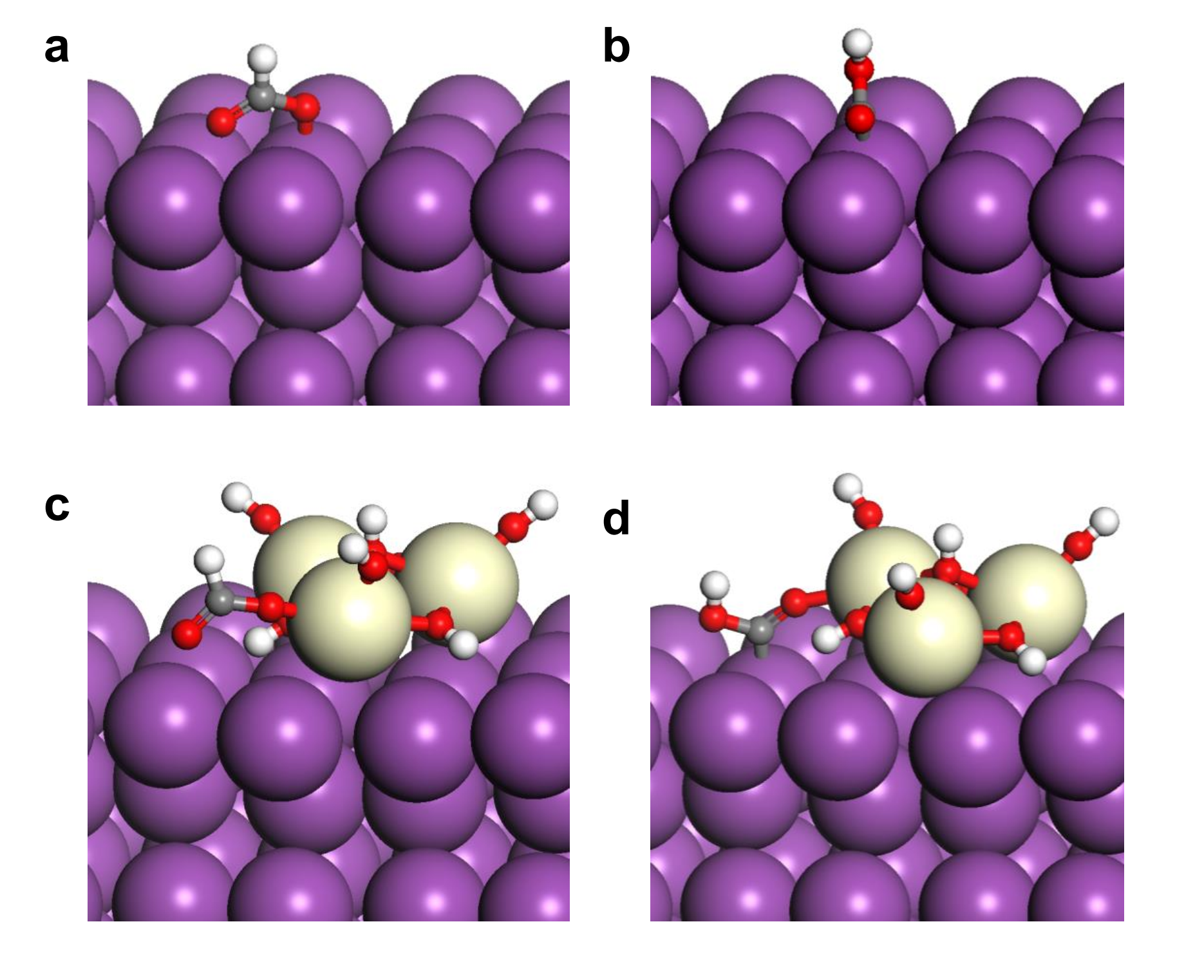


**Supplementary Figure 21.** Optimized configurations of *OCHO (a and c) and *COOH (b and d) intermediates on Bi and CeO_x_/Bi structure models. Purple, yellow, gray, red, and white spheres are Bi, Ce, C, O, and H atoms, respectively.


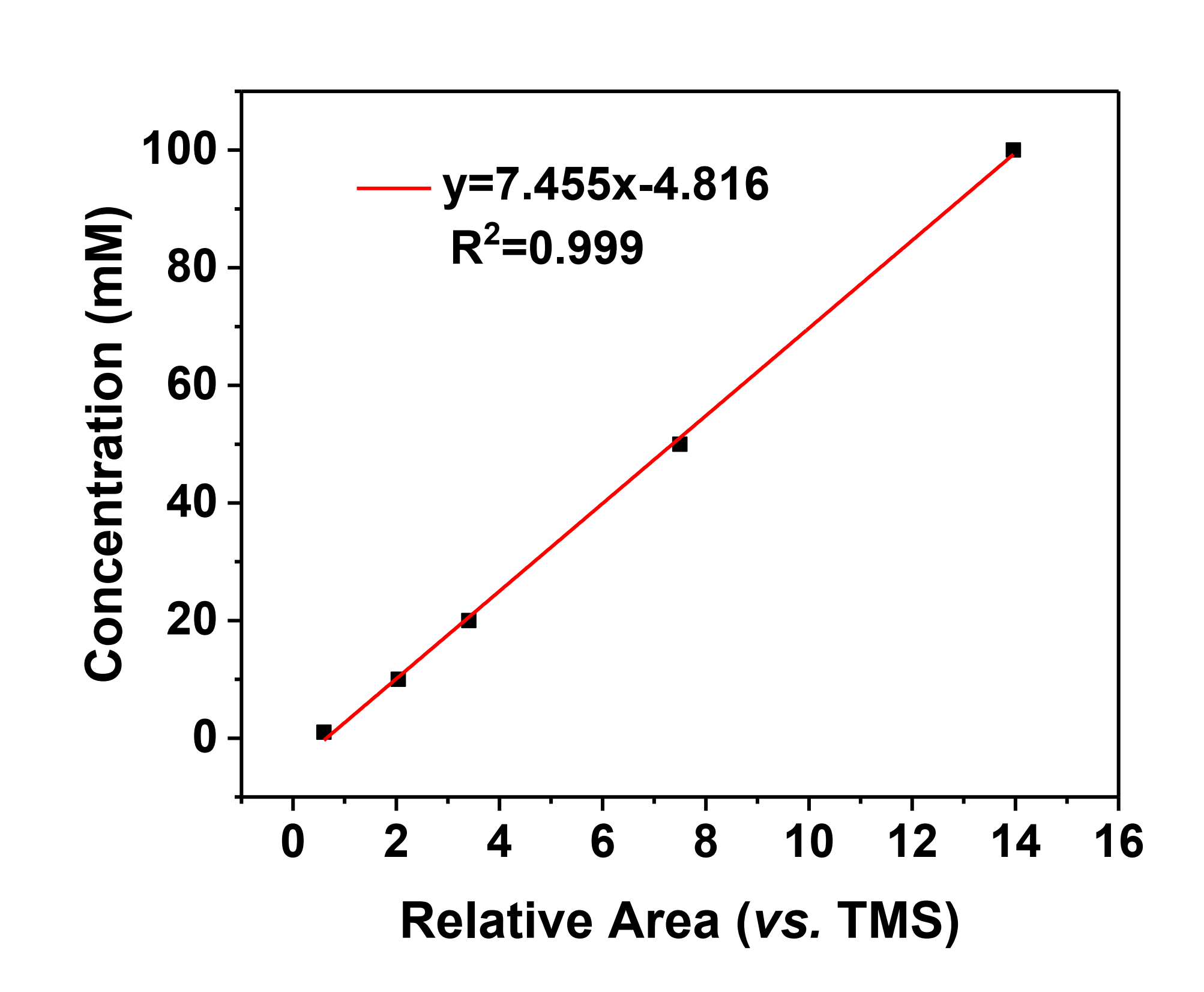


**Supplementary Figure 22.** Calibration curve for standard HCOOK solutions by using 1% tetramethylsilane (TMS) as internal standard.

**Supplementary Table 1.** Summary of Ce 3d XPS results.

| Peaks | Binding energy (eV) | CeO_2_/BiOCl | D-CeO_x_/Bi |
| --- | --- | --- | --- |
| ν_o_ | 880.5 | 218.4654 | 3933.628 |
| ν | 882.5 | 17195.73 | 5613.244 |
| ν′ | 885.3 | 8824.292 | 6860.711 |
| ν′′ | 888.6 | 9351.93 | 2678.534 |
| ν′′′ | 898.3 | 12055.17 | 2538.198 |
| μ_o_ | 899.1 | 2283.62 | 334.4925 |
| μ | 900.8 | 12437.81 | 3281.22 |
| μ′ | 903.7 | 6683.304 | 2997.556 |
| μ′′ | 907.3 | 9220.942 | 2672.017 |
| μ′′′ | 916.7 | 12368.21 | 3376.215 |

Ce^3+^: ν_o_, ν′, µ_o_, µ′′′.

Ce^4+^: ν, ν′′, ν′′′, μ, μ′, μ′′.

CeO_2_/BiOCl: Ce^3+^/(Ce^3+^+Ce^4+^) = 23694.5874/90639.4734 = 0.2614.

D-CeO_x_/Bi: Ce^3+^/(Ce^3+^+Ce^4+^) = 14505.0465/34282.8155 = 0.4231.

**Supplementary references**

1. Li L, Ma D-K and Qi F *et al.* Bi nanoparticles/Bi_2_O_3_ nanosheets with abundant grain boundaries for efficient electrocatalytic CO_2_ reduction. *Electrochim Acta* 2019; **298**: 580-6.
2. Kresse G and Furthmüller J. Efficient Iterative Schemes for Total-Energy Calculations Using a Plane-Wave Basis Set. *Phys Rev B* 1996; **54**: 11169-86.
3. Kresse G and Furthmüller J. Efficiency of ab-initio total energy calculations for metals and semiconductors using a plane-wave basis set. *Comput Mater Sci* 1996; **6**: 15-50.
4. Blöchl P. Projector Augmented-Wave Method. *Phys Rev B* 1994; **50**: 17953-79.
5. Kresse G and Joubert D. From ultrasoft pseudopotentials to the projector augmented-wave method. *Phys Rev B* 1999; **59**: 1758-75.
6. Perdew J and Yue W. Accurate and Simple Density Functional for the Electronic Exchange Energy: Generalized Gradient Approximation. *Phys Rev B* 1986; **33:** 8800-02.
7. Perdew J and Burke K. Ernzerhof, M. Generalized Gradient Approximation Made Simple. *Phys Rev Lett* 1996; **77**: 3865-68.
8. Methfessel M and Paxton A. High-precision sampling for Brillouin-zone integration in metals. *Phys Rev B* 1989; **40**: 3616-21.
9. Dudarev S, Botton G and Savrasov S *et al.* Electron-energy-loss spectra and the structural stability of nickel oxide: An LSDA+U study. *Phys. Rev. B* 1998; **57**: 1505-09.
10. Gao D, Zhang Y and Zhou Z *et al*. Enhancing CO_2_ Electroreduction with the Metal-Oxide Interface. *J Am Chem Soc* 2017; **139**: 5652-55.
11. Nørskov J, Rossmeisl J and Logadottir A *et al.* Origin of the Overpotential for Oxygen Reduction at a Fuel-Cell Cathode. *J Phys Chem B* 2004; **108**: 17886-92.
12. Peterson A, Abild-Pedersen F and Studt F *et al.* How copper catalyzes the electroreduction of carbon dioxide into hydrocarbon fuels. *Energ Environ Sci* 2010; **3**: 1311-15.
13. Luo W, Xie W and Mutschler R *et al.* Selective and Stable Electroreduction of CO_2_ to CO at the Copper/Indium Interface. *ACS Catal* 2018; **8**: 6571-81.
14. Ma W, Xie S and Zhang X et al. Promoting electrocatalytic CO_2_ reduction to formate via sulfur-boosting water activation on indium surfaces. *Nature Commun* 2019; **10**: 892.
